# Sustainable Corrosion Mitigation Using Aqueous Spent Coffee Grounds Extract: Comparative Performance in Different Acidic Media

**DOI:** 10.3390/molecules31162771

**Published:** 2026-08-09

**Authors:** Florina Brânzoi, Denisa-Ioana Răuță (Gheorghe), Roxana-Doina Truşcă, Sorin-Marius Avramescu

**Affiliations:** 1 Institute of Physical Chemistry-Ilie Murgulescu, 202 Splaiul Independentei, 060021 Bucharest, Romania; 2 Biotechnical Systems Engineering Doctoral School, National University of Science and Technology Politehnica Bucharest, 060042 Bucharest, Romania; 3 Faculty of Applied Chemistry and Material Science, National University of Science and Technology Politehnica Bucharest, Splaiul Independentei 313, 060042 Bucharest, Romania; 4 Faculty of Animal Productions Engineering and Management, University of Agronomic, Sciences and Veterinary Medicine of Bucharest, 011464 Bucharest, Romania

**Keywords:** spent coffee grounds, hydrothermal, acidic media, electrochemical analysis, OL 37

## Abstract

This study investigates the efficiency of green corrosion inhibitors derived from spent coffee grounds (SCGs) for OL 37 carbon steel in 0.5 M H_2_SO_4_ and 1 M HCl environments. The aqueous extracts, labeled K1 and K2, were obtained through specialized extraction techniques and characterized by HPLC. Their protective performance was investigated using potentiodynamic polarization and electrochemical impedance spectroscopy (EIS). FT-IR spectroscopy and SEM-EDX analysis confirmed the presence of a protective inhibitor film on the OL 37 surface, attributed to the adsorption of organic molecules from the SCGs extract (K1 and K2). The adsorption behavior followed the Langmuir isotherm, with high adsorption constants and standard free energy values (ΔG°_ads_), indicating a mixed-mode adsorption mechanism. Furthermore, the negative Gibbs free energy values of adsorption confirm the spontaneity of the adsorption process. Thermodynamic studies conducted between 293 K and 333 K demonstrated the temperature dependence of the inhibition process. Results showed that at a concentration of 800 ppm and 1000 ppm, both inhibitors exhibited high efficiency, reaching 96% for K1 and 95% for K2.

## 1. Introduction

Steel corrosion is a serious problem that jeopardizes equipment integrity and security and can potentially result in enormous financial losses. To eliminate oxide deposits from metallic substrates, pickling is a frequently used metallic surface treatment [1]. One common technique used to extend the operating life of steel in the industrial sector is the application of acidic solutions to remove oxides and rust on steel surfaces caused by corrosion. This process is known as pickling, and the most common method is dip pickling [2]. Corrosion causes large financial losses in industries like the oil and gas, as refinery towers, storage tanks, transport pipelines, and other machinery, petrochemical, and automobile sectors [3]. In addition, corrosion results in explosions in chemical factories and the disintegration of metallic structures like buildings, bridges, and overpasses [4].

Investigating corrosion control methods is crucial, and sustainable alternatives are provided by environmentally friendly corrosion inhibitors made from organic ingredients, especially plant extracts. Plant-based extracts are abundant in organic substances like alkaloids and flavonoids that have good corrosion-inhibiting qualities [5,6,7], while strong inhibitors like hydrazine, thiophene, and other inorganic compounds are hazardous to human health and the environment [8,9,10]. They are better alternatives to harmful organic inhibitors since they are non-toxic and biodegradable. Extracts from a variety of plant parts, including fruits, leaves, and seeds, are accessible [11,12,13].

Effective metal corrosion protection methods must therefore be implemented. Implementing corrosion inhibitors appears to be a practical and economical way to mitigate economic losses caused by corrosion [14,15,16,17,18].

Eco-friendly corrosion inhibitors prevent corrosion processes by using π-electronic systems and S, P, N, and O, green inhibitors were adsorbed on the metal surface. They prevent corrosion by adhering to the metal surface either chemically (chemisorption) or physically (physisorption). Inhibitory compounds can be adsorbed chemically or electrostatically by forming a physical or covalent link [14,15]. These inhibitors are advantageous as ecologically friendly corrosion inhibitors because of their non-toxic and biodegradable qualities [19,20,21,22]. Using food waste as an environmentally sustainable resource is a viable substitute for chemical-based compounds. As a result, attempts are being made to create corrosion prevention techniques that follow the values of green chemistry [20,21,22].

Coffee is the second-most traded commodity after oil, grown in more than 70 countries, producing 165–170 million bags annually on average, with a projected USD 36.3 billion export value in 2021 [23,24]. So, it produces a significant volume of spent coffee grounds, annually [25,26].

Due to rising urbanization, expanding coffee culture, and changing consumer tastes, coffee consumption has expanded over the past few decades, placing significant strain on the coffee production system [26,27,28]. Because of this extremely and increasingly high quantity of coffee processed globally, some components, such as caffeine, may be regarded as environmental pollutants, and certain studies are focused on the photocatalytic degradation of such products [29]. Alternatively, in order to reduce the associated environmental effects, the coffee processing sectors are urged to embrace circular economy concepts and put sustainable efforts into action [27,28,30,31]. Spent coffee grounds (SCGs) have attracted considerable attention as a valuable resource for both energy and non-energy applications, thanks to their rich biochemical composition, which includes fatty acids, cellulose, esters, lignin, and hemicelluloses [31,32,33,34].

Numerous studies have been carried out to investigate the corrosion-inhibitory effectiveness of SCG extract in a variety of metals and environmental settings. X70 carbon steel was subjected to a corrosion resistance test in a 0.5 M HCl corrosive solution. As a natural corrosion inhibitor, 100 mL of distilled water mixed with used coffee grounds from a local coffee shop was used. A corrosion inhibition rate of 96% was achieved at 100 ppm [31,32,33,34,35]. It has been reported that hydroethanolic extracts obtained from spent coffee grounds using the Soxhlet method are highly effective corrosion inhibitors on 307 L stainless steel, with an inhibition efficiency of up to 93.5% at 1500 ppm and 298 K [32,33,34,35,36]. Extracts from SCGs were obtained using only ultrapure water and used as corrosion inhibitors for the carbon steel substrate in a 1 mol L^−1^ HCl solution. A maximum efficiency of 94.83% was recorded [30,31,32]. The corrosion of C38 steel was studied in a 1 M HCl solution containing various concentrations of hydroalcoholic extract from used coffee grounds. HECG exhibits high potential as a corrosion inhibitor for C38 steel, with a significant efficiency of 92.71% at a concentration of 2 g L^−1^ and at 323.15 K [34,35,36]. The effects of aqueous extracts from coffee grounds on the corrosion of carbon steel in a 1 mol L^−1^ HCl solution were investigated. Two extraction methods were studied: decoction and infusion. The efficiency of carbon steel corrosion inhibition in a 1 mol L^−1^ HCl solution increased with increasing extract concentration and temperature [35,36,37,38,39].

The novelty of our research lies in the use of aqueous extract from spent coffee grounds (SCGs) as a corrosion inhibitor for OL 37, rolled steel, malleable steel, and mild steel in hydrochloric acid and sulfuric acid environments. A comparison was made of the corrosion inhibition performance of water extracts from SCGs extracts in the two acidic environments, as a function of temperature. To the authors’ knowledge, at the time of writing, no articles with the same objective had been published.

The main objective of this study is to investigate the inhibition of OL 37 corrosion in two acidic solutions, in parallel, using the water-based extract obtained from spent coffee grounds (SCGs) at different inhibitor concentrations and temperatures. To achieve this, several electrochemical techniques were employed, including potentiodynamic polarization, and electrochemical impedance spectroscopy (EIS).

## 2. Results and Discussion

### 2.1. FT-IR

**Figure 1:**
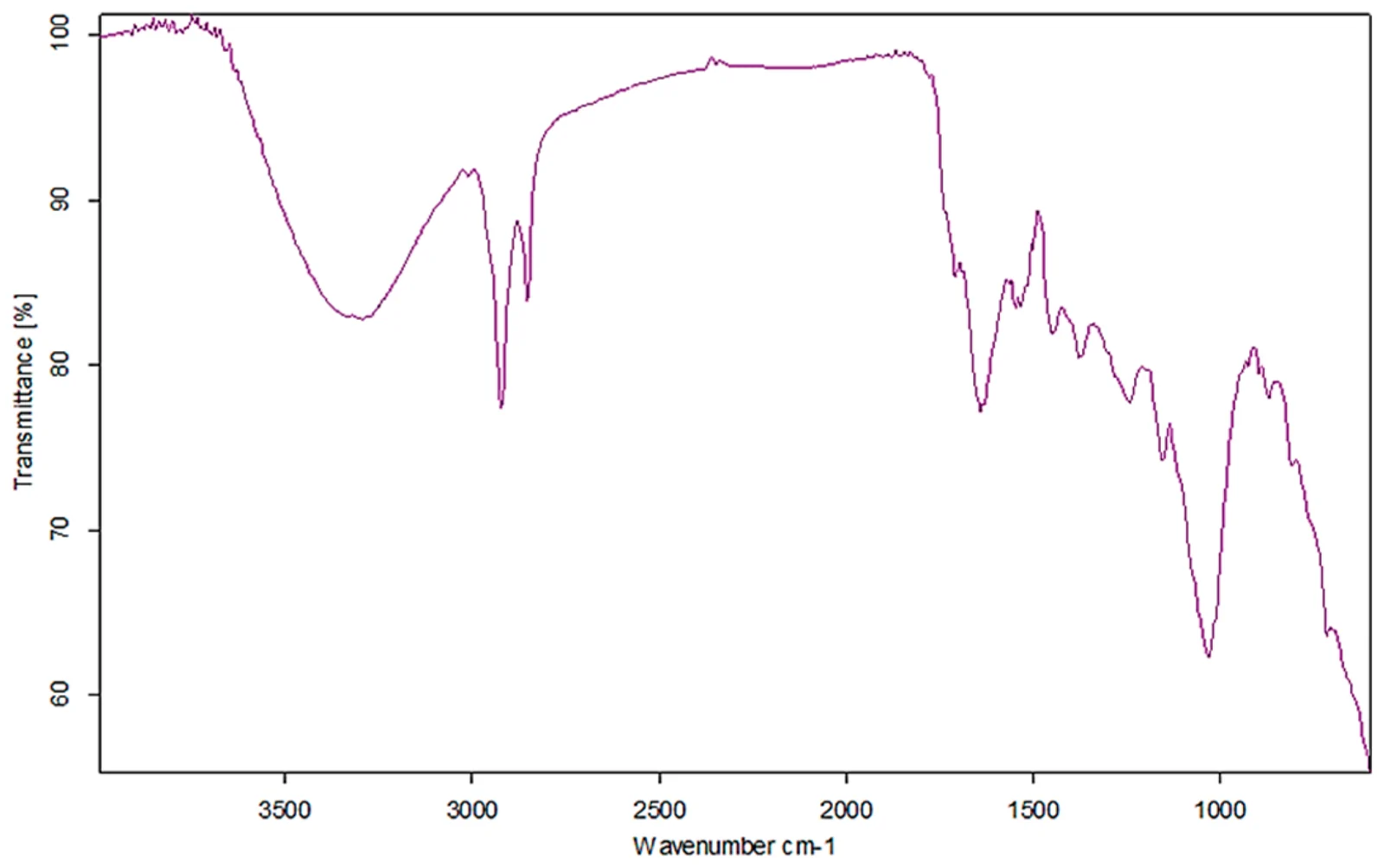
FT-IR spectrum SCG powder.

FT-IR spectroscopic analysis (Figure 1) indicated that the broad band observed at 3366 cm^−1^ was attributed to the stretching of the O-H group due to inter- and intramolecular hydrogen bonding of polymeric compounds such as alcohols, phenols, and carboxylic acid in pectin, cellulose, and lignin. The O-H stretching vibrations appear over a wide range of frequencies, indicating the presence of free hydroxyl groups and O-H bands associated with carboxylic acids. The band at 2925 cm^−1^ indicates the symmetric or asymmetric C-H stretching vibration of aliphatic acids. The absorption peaks at 1745 cm^−1^ were attributed to the resulting carboxyl bond, derived from xanthine derivatives such as caffeine.

### 2.2. HPLC Analysis of SCG

**Figure 2:**
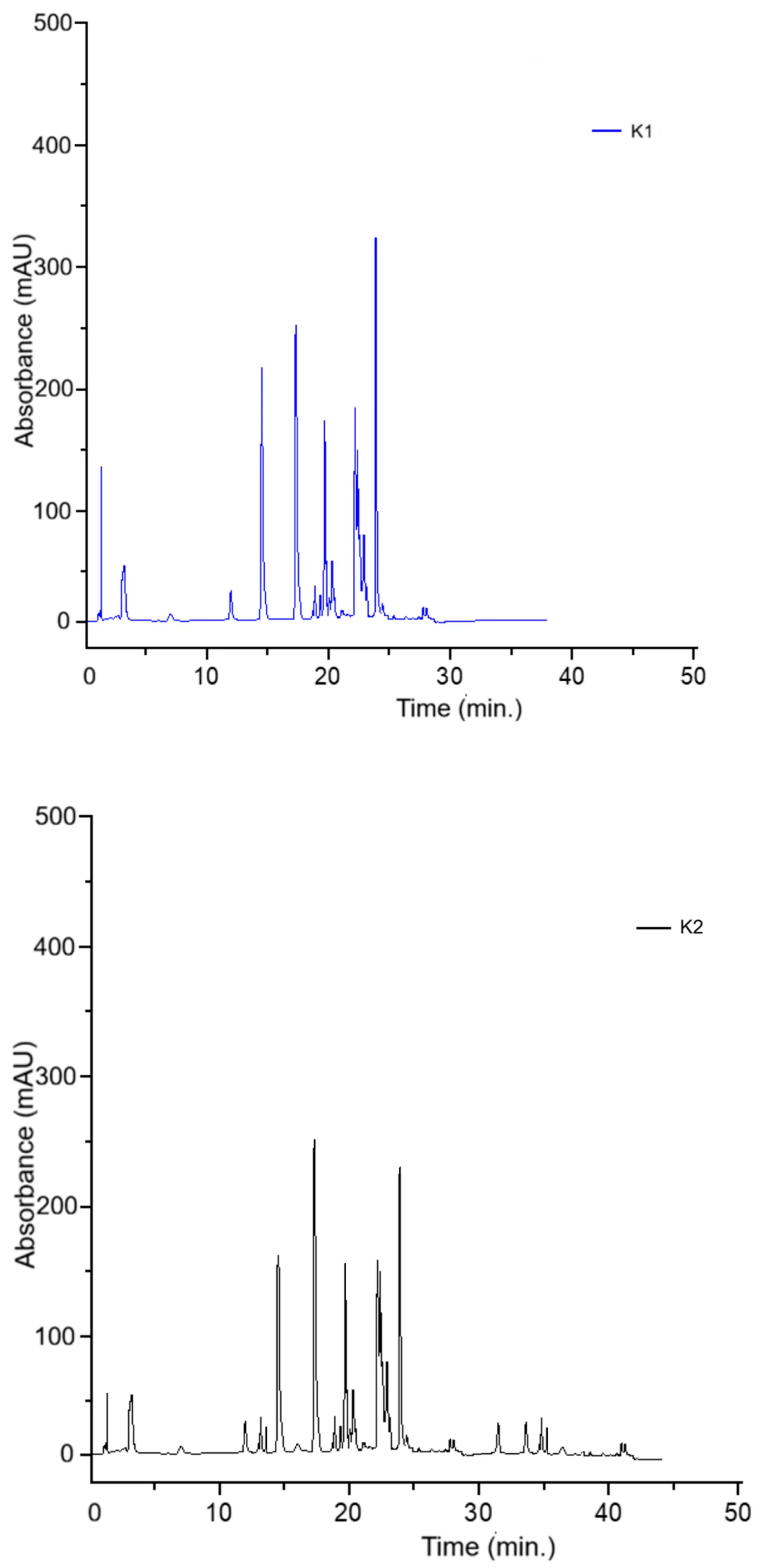
HPLC chromatogram of the aqueous extract of used coffee grounds.

The used coffee grounds, extracted by the hydrothermal method, were subjected to HPLC analysis, and the obtained chromatogram is represented in Figure 2 at the selected wavelength of 280 nm, which is the most relevant for visualizing the chromatographic peaks. The hydrothermal extraction temperature had a major influence on the phenolic profile of the aqueous extracts obtained from coffee grounds (SCGs). Increasing the extraction temperature from 150 °C to 220 °C resulted in significant quantitative changes in the concentration of individual phenolic compounds, due to the combined effects of enhanced mass transfer, hydrolysis reactions, thermal degradation, decarboxylation, and the conversion of high-molecular-weight polyphenols into simpler phenolic derivatives.

At 150 °C, chlorogenic acid was identified as one of the dominant phenolic constituents, along with syringic acid and caffeic acid. When the extraction temperature was increased to 220 °C, a substantial decrease in the concentration of chlorogenic acid was measured. At higher temperatures, chlorogenic acid undergoes thermal degradation, isomerization and hydrolysis, generating supplementary quantities of caffeic acid (and quinic acid), consequently increasing its total concentration (Table 1). The increase in gallic acid concentration at high temperatures can be attributed to the hydrolysis of gallate structures and condensed polyphenolic compounds present in the lignocellulosic matrix of spent coffee grounds. Extraction with water at higher temperatures promotes the breaking of hydrogen bonds and improves solvent penetration into the biomass matrix, facilitating the release of bound phenolic compounds.

Syringic acid had the highest concentration among the identified phenolic compounds, and its content increased even further at 220 °C as a result of the thermal degradation of lignin-derived aromatic structures. In contrast, catechin and epicatechin are extremely sensitive to thermal oxidation and polymerization reactions under hydrothermal conditions, which is the reason for their severe concentration decrease for the 220 °C sample (i.e., K_2_). Caffeine exhibits relatively high thermal stability compared to phenolic compounds Therefore, the slight increase in concentration observed for the 220 °C sample is most probably due to increased extraction efficiency and diffusion phenomena. The results indicate that extraction at 220 °C promotes the conversion of complex polyphenolic constituents into lower-molecular-weight phenolic acids, leading to a phenolic profile enriched with thermally induced reaction products, such as caffeic, syringic acid, and ferulic acid, while simultaneously reducing the concentration of thermolabile constituents, including chlorogenic acid, catechin, and epicatechin. These findings demonstrate that extraction temperature is a critical parameter that controls both the yield and the chemical composition of aqueous SCG extracts, directly influencing their potential anticorrosive properties.

The extract obtained at 220 °C is expected to exhibit increased antioxidant activity due to the higher concentration of low-molecular-weight phenolic acids generated by the thermally generated chlorogenic acid derivatives. However, the extract obtained at 150 °C may retain a higher proportion of complex-structured polyphenols, capable of forming more stable adsorbed protective layers on metal substrates. Therefore, while the extract at 220 °C may offer superior radical scavenging performance, the extract at 150 °C could exhibit comparable or even improved corrosion inhibition efficiency.

In the extract obtained at 150 °C, the polyphenolic compounds are less degraded and retain their complex aromatic structures, which promote adsorption onto the steel surface. The higher concentrations of chlorogenic acid, catechin, and epicatechin are particularly important because these molecules contain multiple hydroxyl groups and π-conjugated systems capable of interacting with the vacant d orbitals of the iron atoms on the OL 37 surface.

In contrast, at 220 °C, significant thermal degradation of complex polyphenols occurs, and the extract becomes dominated by smaller molecules, such as caffeic acid and phenolic acid derivatives, which can lead to a decrease in surface coverage and a reduction in the ability to form a compact and stable layer.

### 2.3. Electrochemical Analysis

#### 2.3.1. Potentiodynamic Polarization Studies

**Figure 3:**
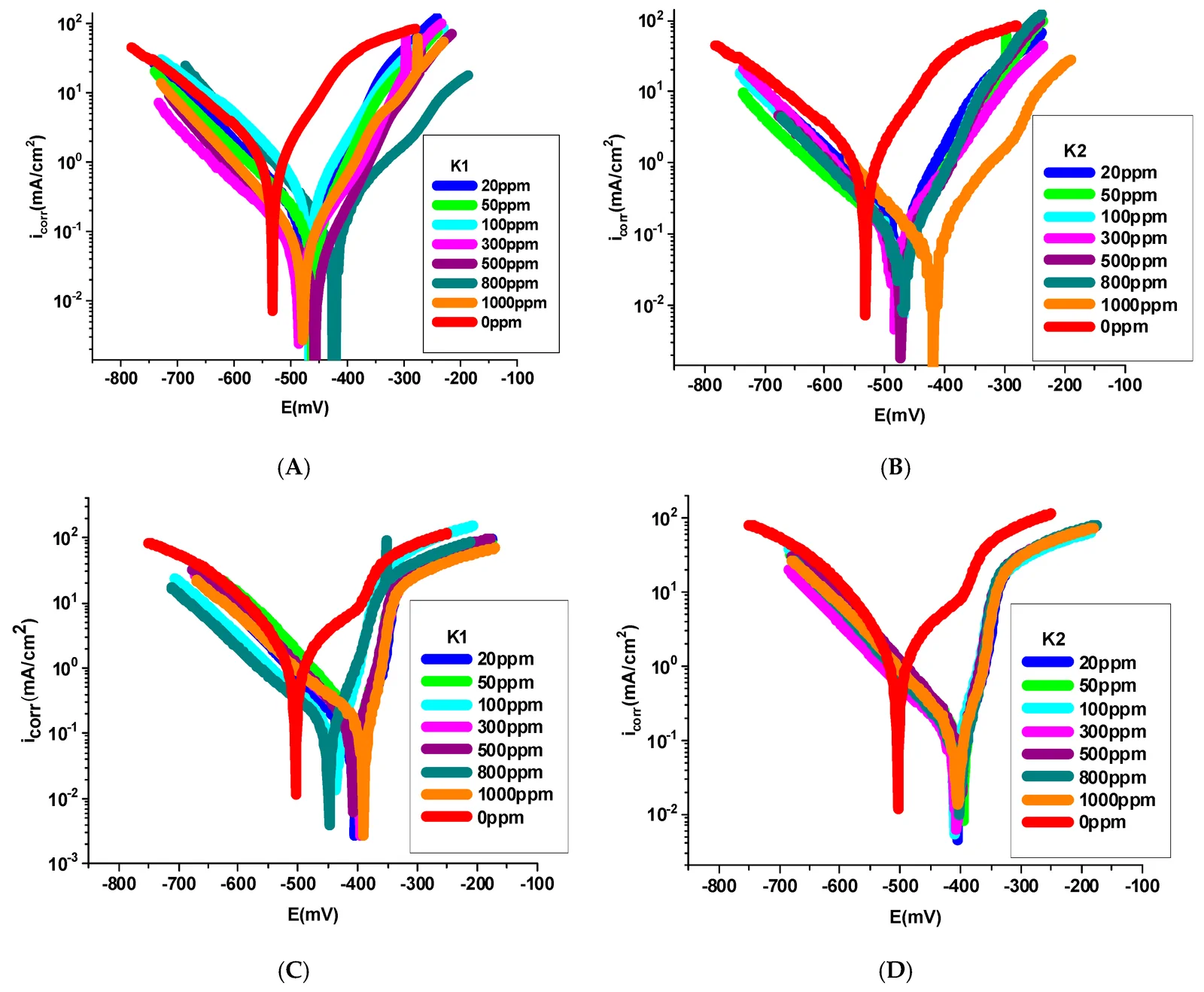
Potentiodynamic polarization curves of OL 37 carbon steel in 1 M HCl (**A**,**B**) and 0.5 M H_2_SO_4_ (**C**,**D**) solutions in the absence and presence of different concentrations of the corrosion inhibitors K1 and K2 at 25 °C.

This study explores the application of SCG-derived green corrosion inhibitors as an efficient strategy for safeguarding OL 37 carbon steel under aggressive conditions (sulfuric and hydrochloric acid media), targeting anodic, cathodic, or mixed-type corrosion mechanisms. The inhibitory performance of K1 and K2 was assessed in 0.5 M H_2_SO_4_ and in 1 M HCl using potentiodynamic polarization and electrochemical impedance spectroscopy (EIS). Surfaces treated with the spent coffee ground (SCG) extracts exhibited a significant decrease in both anodic and cathodic current densities, demonstrating effective suppression of the underlying electrochemical reactions. The polarization curves indicate that current densities in the presence of K1 and K2 are substantially lower than those of the uninhibited system. This behavior identifies the extracts as mixed-type inhibitors that impede both anodic metal dissolution and cathodic hydrogen evolution. Furthermore, the addition of these green compounds shifts the corrosion potential (E_corr_) toward more noble (positive) values compared to the uninhibited sample, indicating a predominant influence on the anodic process. Figure 3 illustrates the potentiodynamic polarization curves for OL 37 specimens immersed in 0.5 M H_2_SO_4_ and 1 M HCl solutions, comparing the uninhibited environments with those containing the green inhibitors (K1 and K2).

The electrochemical parameters—including corrosion current density (i_corr_), corrosion potential (E_corr_), anodic/cathodic Tafel slopes (b_a_, b_c_), open circuit potential (OCP) and inhibition efficiency (E%)—are presented in Table 2, Table 3, Table 4 and Table 5. These data indicate that the addition of the spent coffee ground (SCG) extract shifts the OCP to more positive values relative to the uninhibited solution. This behavior can be attributed to the adsorption of the SCG extract onto the OL 37 electrode surface, which forms a protective adsorbed layer. Based on the data presented in Figure 3 and Table 2, Table 3, Table 4 and Table 5, a clear decrease in the corrosion current densities is observed with increasing concentrations of the green inhibitors K1 and K2 in the acidic media. This inhibitory effect is likely governed by the competitive adsorption of inhibitor molecules and/or aggressive ions (Cl^−^ and SO_4_^2−^) onto the active anodic zones of the OL 37 carbon steel surface, thereby suppressing the oxidation reaction. Furthermore, the experimental results confirm that increasing concentrations of K1 and K2 significantly minimize the corrosion current density while enhancing the protection efficiency. Overall, these results demonstrate the formation of a protective molecular film on the OL 37 steel surface, effectively deactivating the active corrosion centers. A comparative evaluation of the inhibition performance and corrosion kinetics, quantified through corrosion rates (Rmpy, in mil/year; P, in mm/year; and Kg, in g m^−2^ h^−1^), demonstrates that both eco-friendly compounds, K1 and K2, provide effective anticorrosive protection for the OL 37 substrate in 1 M HCl and 0.5 M H_2_SO_4_ environments. This defensive mechanism is governed by the adsorption of inhibitor molecules onto the active surface sites, accompanied by the formation of a stable interfacial film on the OL 37 steel surface.

As illustrated in Figure 3 and Table 2, Table 3, Table 4 and Table 5, the optimum protection performance for the K1/OL 37 and K2/OL 37 systems in HCl medium was achieved at concentrations of 800 ppm and 1000 ppm, respectively, whereas in the H_2_SO_4_ medium, the highest inhibition efficiencies were obtained at 1000 ppm and 800 ppm, respectively. Although K1 exhibited a slightly higher inhibition efficacy (96%) than K2 (95%) at concentrations of 800 ppm and 1000 ppm in 1 M HCl, both extracts produced nearly identical protection efficiencies (95%) at 800 ppm and 1000 ppm in 0.5 M H_2_SO_4_. This minor difference falls within the limits of experimental error. Consequently, both green extracts (K1 and K2) demonstrate comparable inhibitory performance under the investigated conditions. Nevertheless, this slight discrepancy may be tentatively attributed to the higher concentration of polyphenolic compounds present in the green inhibitor, as confirmed by the HPLC data presented in Table 1. These organic constituents contain various functional groups (e.g., –OH, –COOH, and aromatic rings) as well as heteroatoms (O, N, and S). The π-electron-rich centers associated with these moieties facilitate strong interactions with the metal surface through either physisorption or chemisorption mechanisms. Furthermore, a synergistic effect among the major components of the SCG extracts likely enhances their adsorption onto the OL 37 substrate, thereby promoting the formation of a stable and resilient protective layer. This competitive adsorption process blocks the active surface sites, effectively shielding the OL 37 substrate from aggressive corrosive species (Cl^−^ and SO_4_^2−^). Regarding the inhibition mechanism, adsorption processes dominate at lower inhibitor concentrations, leading to a sharp increase in inhibition efficiency, whereas surface saturation at higher concentrations results in a characteristic performance plateau.

#### 2.3.2. Influence of Immersion Time

**Figure 4:**
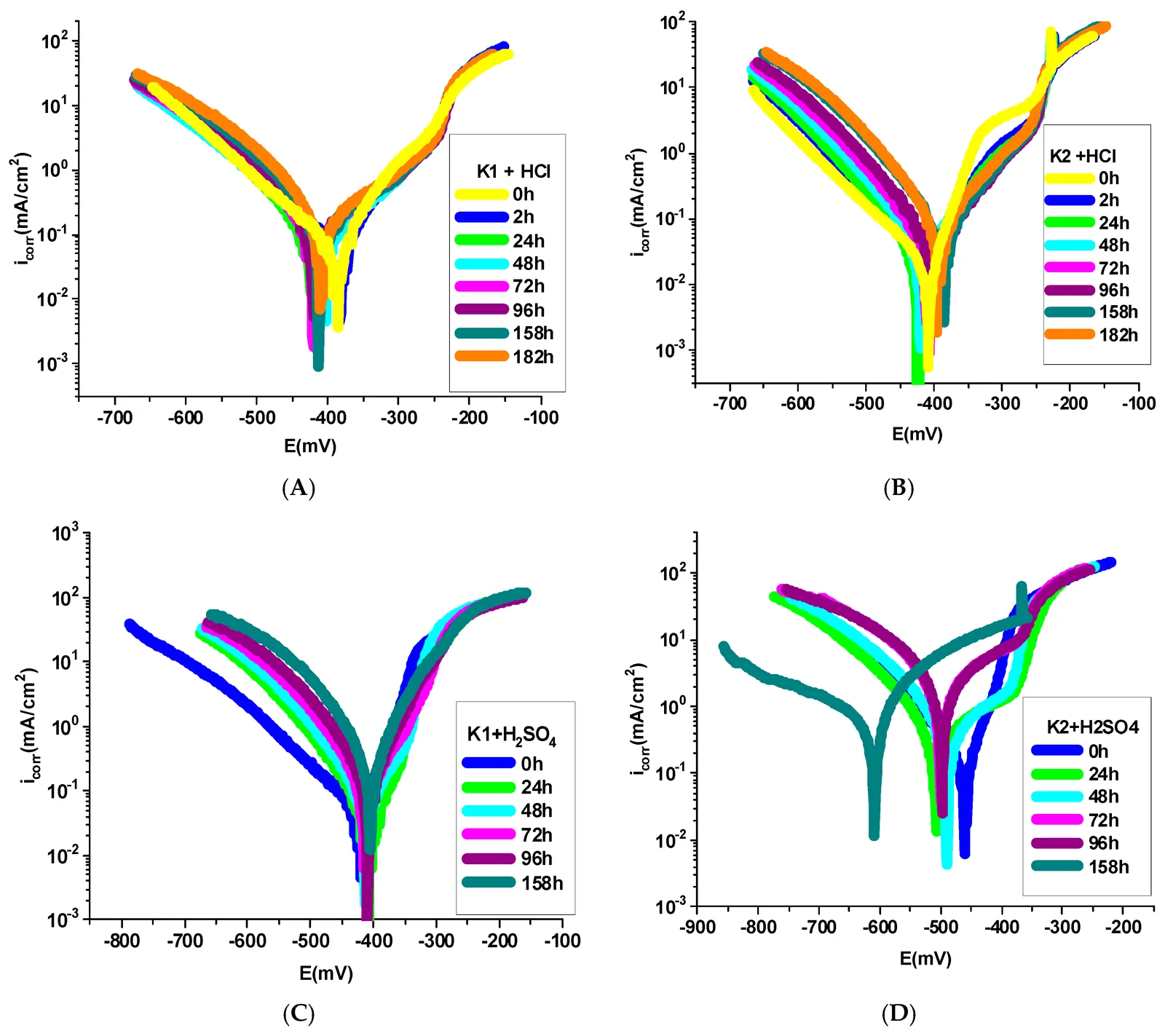
Potentiodynamic polarization curves of OL 37 carbon steel in 1 M HCl (**A**,**B**) and 0.5 M H_2_SO_4_ (**C**,**D**) solutions at various immersion times in the presence of 1000 ppm of the corrosion inhibitors K1 and K2 at 25 °C.

Potentiodynamic polarization was employed to evaluate the impact of extending the immersion time (from 0 to 158 h) on the anti-corrosive performance of organic compounds K1 and K2 (1000 ppm) on OL 37 carbon steel in 1 M HCl and 0.5 M H_2_SO_4_. The time-dependent evolution of the protective efficacy of these inhibitors is illustrated in Figure 4A–D and detailed in Table 6, Table 7, Table 8 and Table 9. Notably, even after 96 h of exposure, the inhibition efficiency in 1 M HCl remains remarkably high at 88% for K1 and 90% for K2 (Figure 4A,B and Table 6 and Table 7), confirming their outstanding stability and effectiveness over extended periods. This sustained durability in the aggressive medium further substantiates the excellent protective capacity of both extracts. Despite this, even after 96 h of immersion in 0.5 M H_2_SO_4_ the inhibition efficiency remains high at 88% for K1, whereas for K2 it drops to 68% (Figure 4 C,D and Table 8 and Table 9). This demonstrates that while K1 maintains highly effective inhibition over extended exposure periods, the protective performance of K2 in 0.5 M H_2_SO_4_ gradually diminishes over time. Indeed, a distinct increase in the corrosion rate is observed for K2 after 158 h of immersion (Figure 4D and Table 9). This behavior is attributed to the progressive degradation of the K2 inhibitory layer during prolonged exposure, resulting from changes within the active zone. This degradation may be driven by specific defects within the adsorbed film that facilitate the penetration of aggressive sulfate ions (SO_4_^2−^) to the OL 37 inhibitor interface. Consequently, while K1 provides robust, long-term corrosion protection under both acidic conditions, K2 exhibits a more time-limited efficacy in sulfuric acid environments.

#### 2.3.3. Electrochemical Impedance Spectroscopy (EIS) Analysis

**Figure 5:**
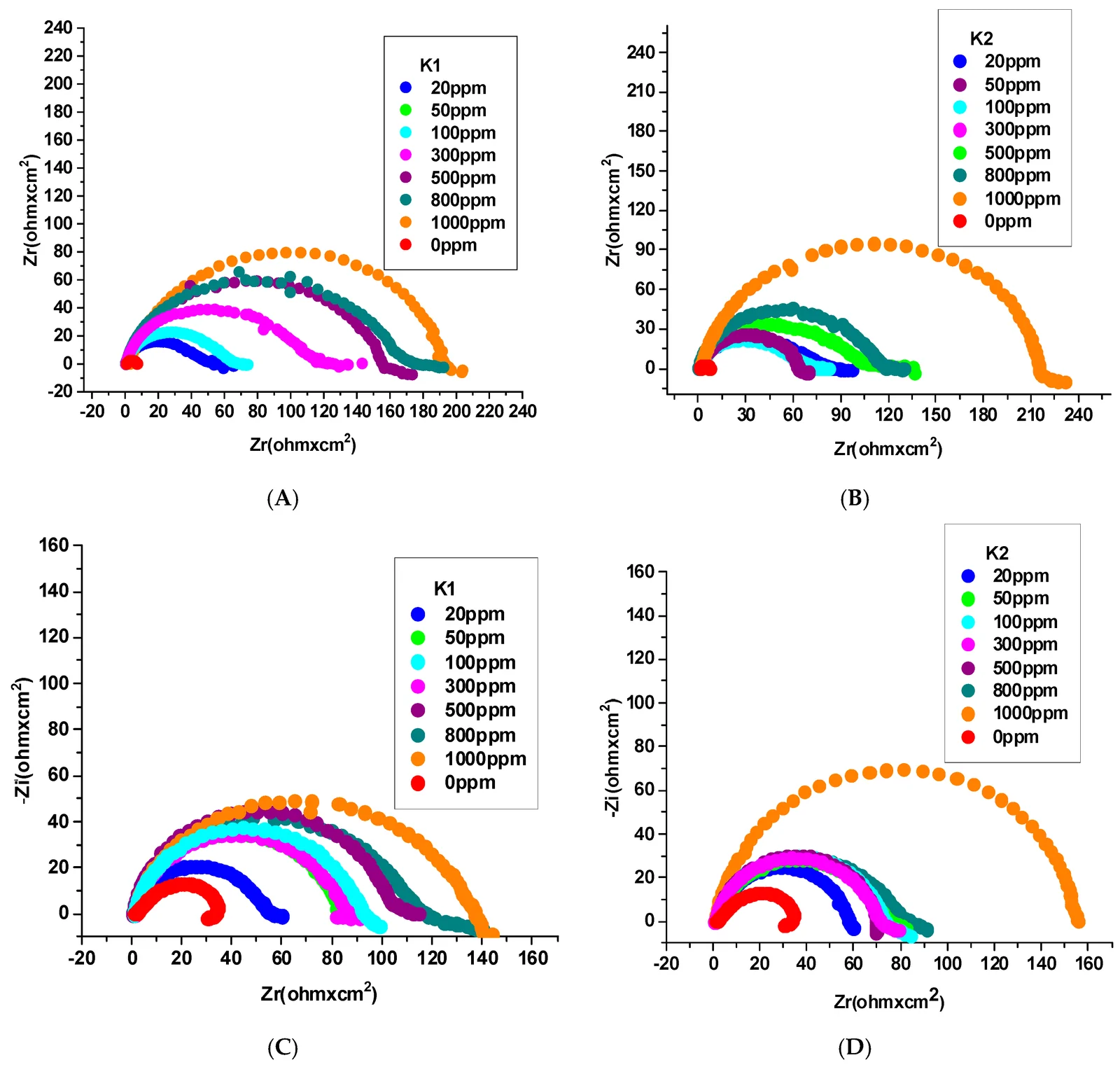
Nyquist plots obtained for OL 37 carbon steel in 1 M HCl (**A**,**B**) and 0.5 M H_2_SO_4_ (**C**,**D**) solutions in the absence and presence of different concentrations of the corrosion inhibitors K1 and K2 at 25 °C.

Electrochemical impedance spectroscopy (EIS) was employed to evaluate the corrosion inhibition efficiency of green inhibitors derived from spent coffee grounds (SCGs) on OL 37 carbon steel in 1 M HCl and 0.5 M H_2_SO_4_ media. The EIS data confirmed the anticorrosive efficacy of the SCG extracts (K1 and K2) on the OL 37 substrate when exposed to these aggressive environments. Furthermore, the impedance parameters provided valuable insights into the inhibition mechanism, indicating the formation of a protective film on the metal surface. The Nyquist plots for the OL 37 carbon steel at the metal/electrolyte interface, both in the absence and presence of varying inhibitor concentrations, are displayed in Figure 5.

As shown in Figure 5, the uninhibited OL 37 sample displays a small capacitive loop in the Nyquist plots, indicating that the corrosion process is predominantly governed by charge transfer. Upon the addition of the green corrosion inhibitors (SCG), these capacitive loops expand significantly while maintaining their characteristic shape, which is typical for charge transfer-controlled mechanisms. The plots generally exhibit a single time constant, corresponding to the charge transfer reaction at the metal/solution interface as modulated by the adsorption of bioactive components. Furthermore, the diameter of these loops is markedly larger for the inhibited specimens compared to the blank OL 37 electrode and increasing progressively with higher SCG concentrations. This trend reflects a substantial rise in charge transfer resistance, thereby confirming the enhanced corrosion protection provided by the SCG extracts for the OL 37 substrate in both 1 M HCl and 0.5 M H_2_SO_4_ media. The Nyquist plots demonstrate that the addition of SCG significantly alters the impedance response of the OL 37 carbon steel, verifying the development of a protective barrier in the presence of both K1 and K2. As displayed in Figure 5, the impedance diagrams deviate from ideal semicircular behavior, a phenomenon typically attributed to frequency dispersion caused by the surface roughness and structural inhomogeneities of the OL 37 substrate. Notably, the diameters of the capacitive loops obtained at 800 and 1000 ppm for K1 and K2 are substantially larger than those of the uninhibited sample. This enlargement reflects a remarkable enhancement in charge transfer resistance, further confirming that these spent coffee ground (SCG) extracts function as highly efficient green inhibitors, for OL 37 carbon steel in these aggressive acidic environments.

**Figure 6:**
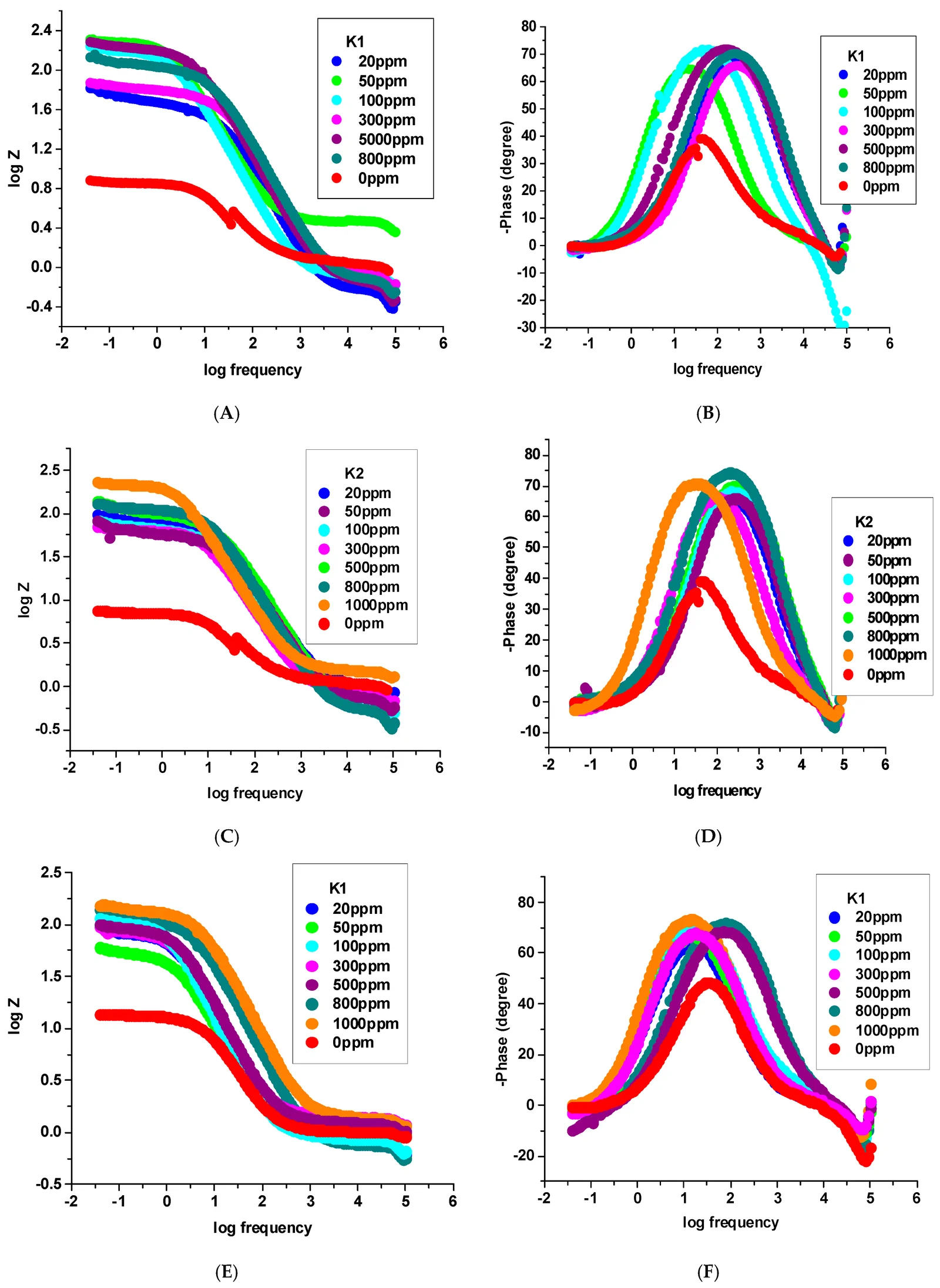

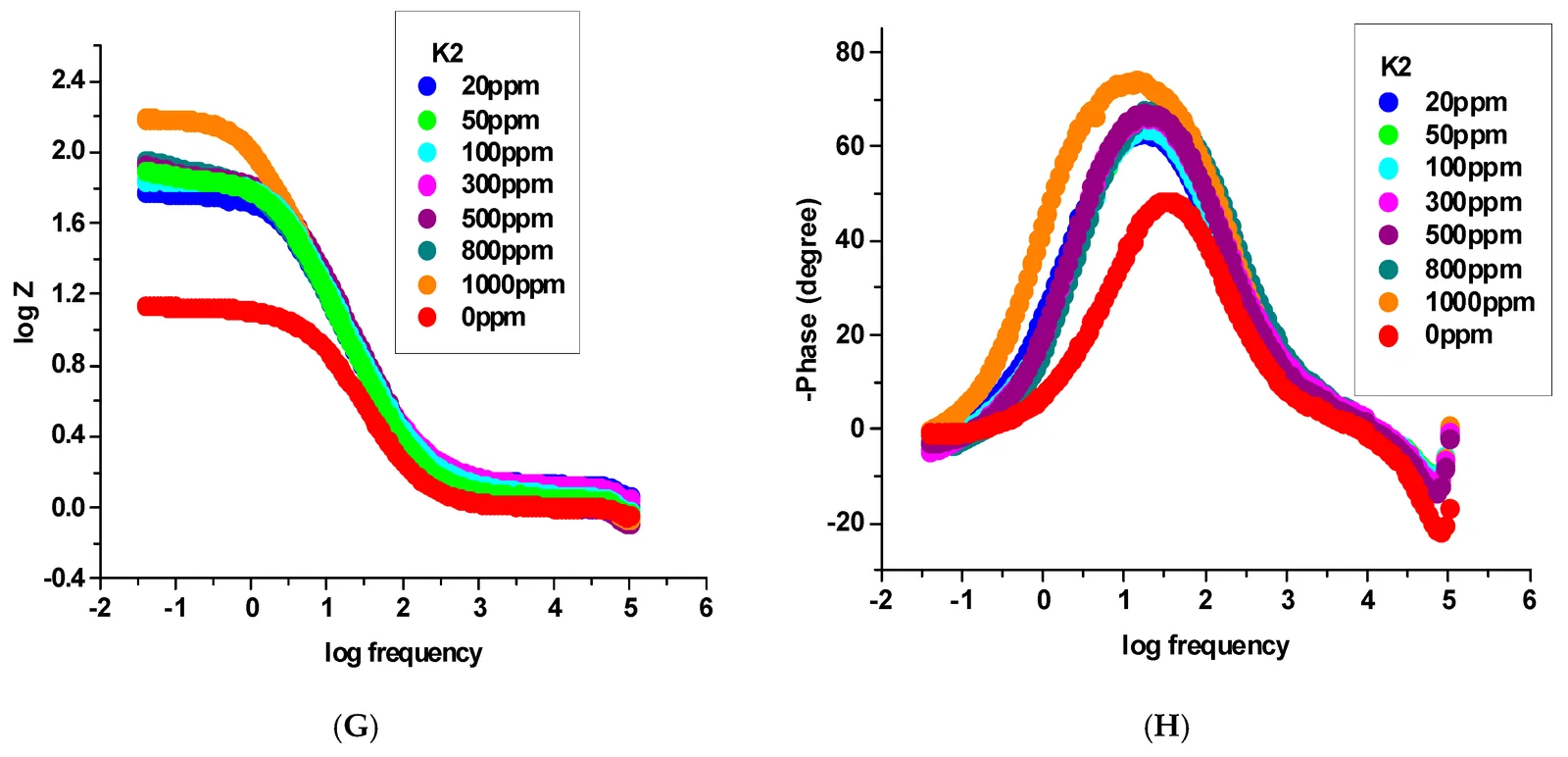
Bode plots obtained for OL 37 carbon steel in 1 M HCl (**A**–**D**) and 0.5 M H_2_SO_4_ (**E**–**H**) solutions in the absence and presence of different concentrations of the corrosion inhibitors K1 and K2 at 25 °C.

**Figure 7:**
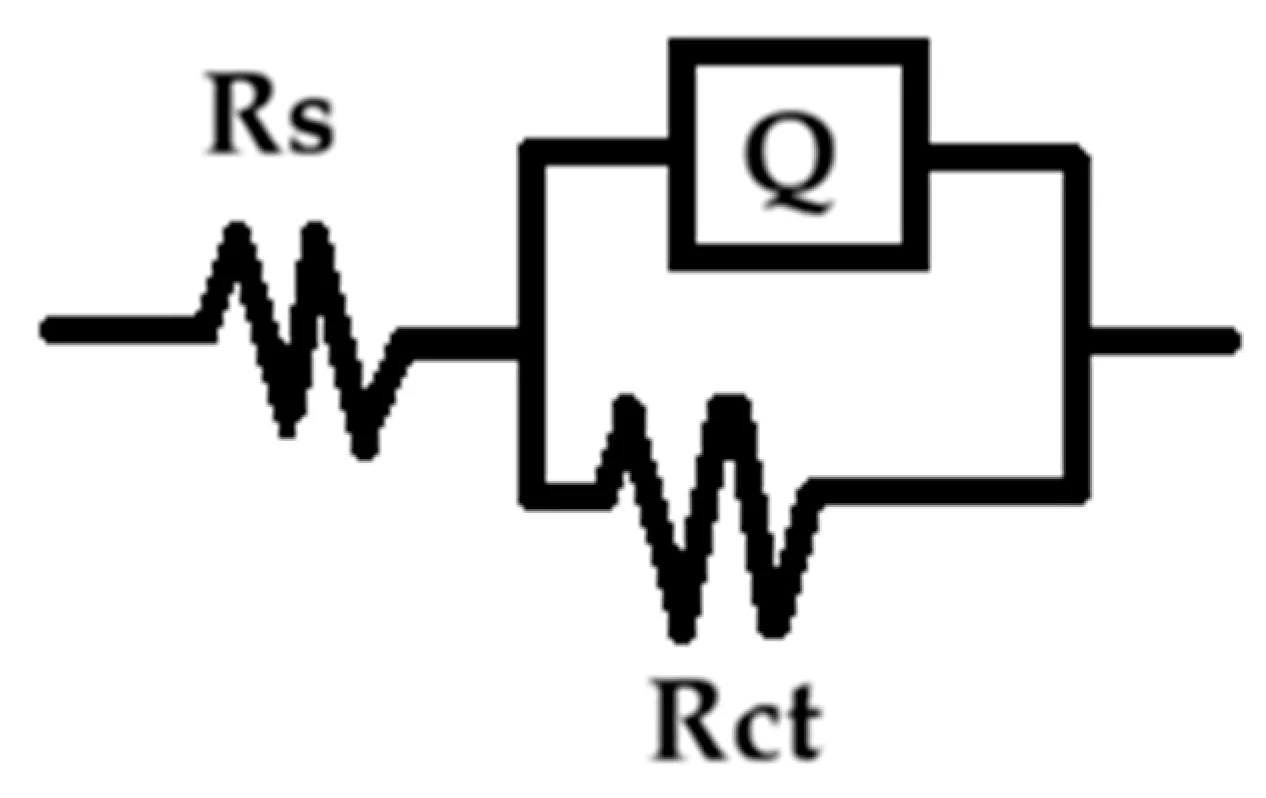
Equivalent electrical circuit model of the studied system.

As illustrated in the Bode plots (Figure 6), the impedance modulus at low frequencies rises progressively with higher inhibitor concentrations. This trend indicates that the adsorption of phytochemical constituents enhances the corrosion resistance of the OL 37 substrate within the acidic electrolytes. The presence of a single time constant suggests that electrolyte penetration to the metal surface is restricted, thereby significantly suppressing the corrosion process. For the uninhibited OL 37 sample, the phase angle reaches approximately 40–48° at medium-to-low frequencies, reflecting a non-ideal capacitive response often linked to surface roughness or weak diffusion. Upon the addition of the SCG extracts (K1 and K2), the phase angle plots (phase angle vs. log frequency) exhibit a well-defined maximum shifting toward roughly 70–75°. This notable increase indicates a transition to a more pronounced capacitive behavior, confirming the formation of a stable protective film on the OL 37 substrate. Consequently, the inhibited specimens display enhanced capacitive properties, aligning perfectly with the findings from the Nyquist plots and potentiodynamic polarization curves. The overall increase in |Z| with higher SCG concentrations directly correlates with improved inhibition efficiency and superior protective performance. To model the electrochemical behavior, the experimental EIS spectra were fitted using an R(QR) equivalent electrical circuit, as depicted in Figure 7. This configuration aligns with the single time constant previously identified in the Bode diagrams. The extraction of key electrochemical parameters specifically, solution resistance (R_s_), charge transfer resistance (R_ct_), and double-layer capacitance (C_dl_), was based on this circuit model, with the results compiled in Table 10, Table 11, Table 12 and Table 13.

To account for the non-ideal capacitive response of the system, a constant phase element Q (CPE) was integrated into the circuit instead of a pure capacitor. The use of a CPE addresses the depression of the capacitive loop, a phenomenon typically caused by surface heterogeneity resulting from substrate roughness and microscopic defects. The impedance of the CPE (Z_CPE_) is mathematically expressed as follows:

Z_CPE_ = Y_0_^−1^ (jω)^−n^

where Z_CPE_ represents the CPE impedance, ω denotes the angular frequency, j is the imaginary unit (j^2^ = −1), Y_0_ is the CPE magnitude (admittance factor), and n is the deviation exponent (phase shift). The empirical exponent *n* serves as a measure of the substrate’s surface inhomogeneity. A higher “n” value indicates a smoother surface with reduced heterogeneity (i.e., fewer structural imperfections). Depending on the value of n, the CPE simplifies to standard electrical components: it acts as a pure resistor when n = 0, (Y_0_ = R), an ideal capacitor when n = 1 (Y_0_ = C), and an inductor when n = −1 (Y_0_ = 1/L). EIS results indicated that the inhibition of the OL 37 substrate by K1 and K2 induces a progressive increase in charge transfer resistance (R_ct_) alongside a reduction in double-layer capacitance (C_dl_). The upward trend of R_ct_ with rising SCG concentrations indicates a significant improvement in the protective barrier, confirming the robust anticorrosive performance of these eco-friendly inhibitors on OL 37 carbon steel. This behavior is fundamentally ascribed to the adsorption of the inhibitor molecules at the metal/electrolyte interface. This adsorption process displaces water molecules, thereby reducing the local dielectric constant and/or increasing the thickness of the electrical double layer, which ultimately lowers the C_dl_ values. The phytochemical constituents within the SCG extract adsorb onto the OL 37 surface to form a dense, protective film. Furthermore, the combined Nyquist and Bode plots illustrate that these eco-friendly formulations suppress corrosion by acting as an effective diffusion barrier and preventing charge transfer reactions at the interface.

**Figure 8:**
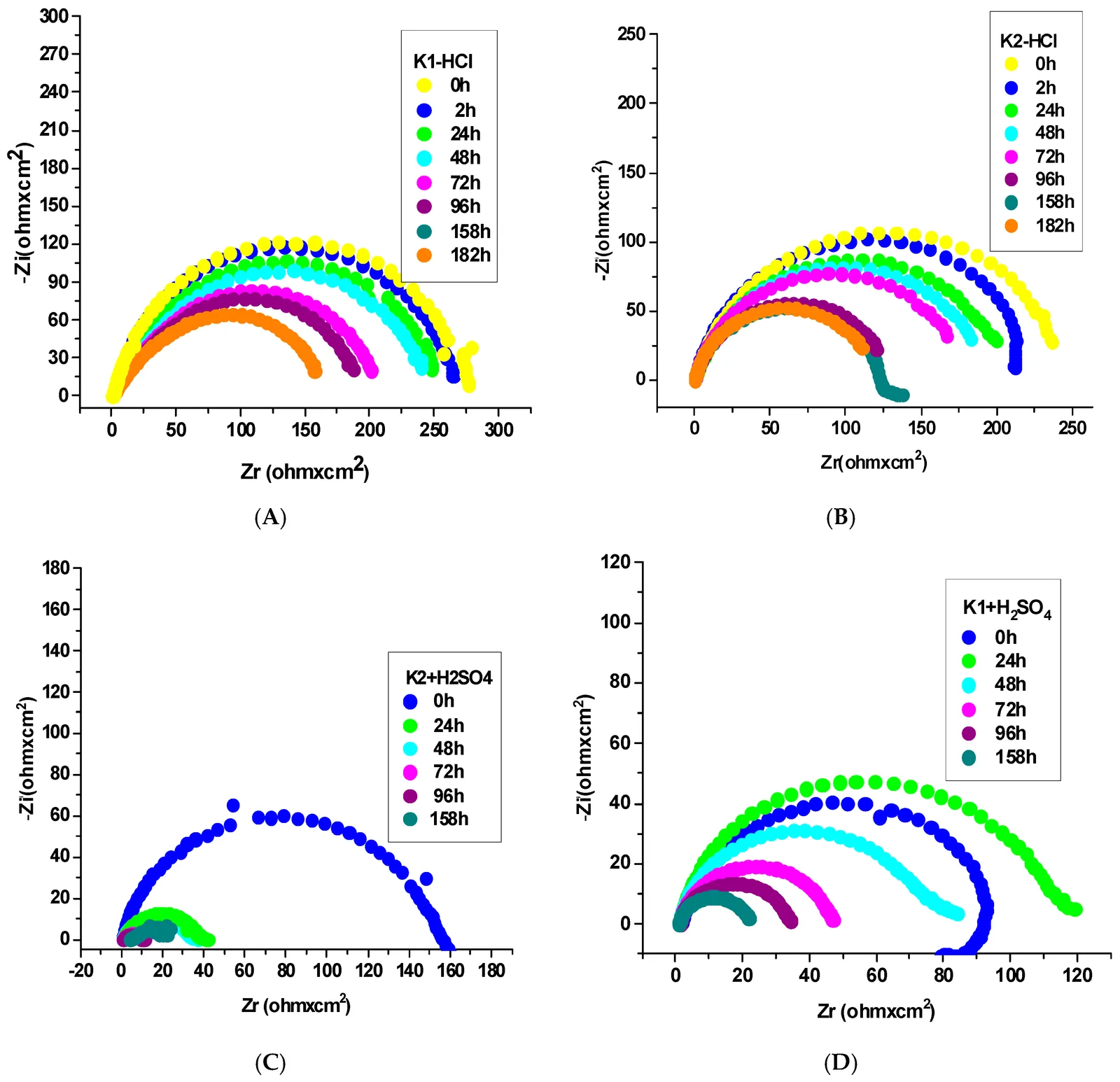
Nyquist plots of OL 37 carbon steel in 1 M HCl (**A**,**B**) and 0.5 M H_2_SO_4_ (**C**,**D**) of OL 37 carbon steel in 1 M HCl (**A**,**B**) and 0.5 M H_2_SO_4_ (**C**,**D**) solutions at various immersion times in the presence of 1000 ppm of the corrosion inhibitors K1 and K2 at 25 °C.

The long-term corrosion inhibition performance of the eco-friendly inhibitors K1 and K2 (at 1000 ppm) on OL 37 steel was evaluated using Electrochemical Impedance Spectroscopy (EIS) over an extended immersion period (0–158 h) in 1 M HCl and 0.5 M H_2_SO_4_. The time-dependent protective capacity of these inhibitors is displayed in Figure 8. As seen in Figure 8, the diameter of the capacitive loops decreases slightly over time for both K1 and K2. Consequently, a minor reduction in the inhibition efficiency of the film is observed between 0 and 158 h of immersion. This decline is likely caused by the diffusion of aggressive chloride (Cl^−^) and sulfate (SO_4_^2−^) ions through the protective layer. The EIS data presented in Figure 8A,B confirm that these green inhibitors successfully impede the migration of corrosive chloride ions (Cl^−^) to the metal surface over prolonged exposures. Notably, after 96 h of immersion, the inhibition efficiency of K1 and K2 remains stable, demonstrating their durability and effectiveness as a long-term corrosion inhibitor. Its non-degradable nature within the aggressive environment further substantiates their excellent protective ability. Conversely, as shown in Figure 8C,D, the performance of K2 begins to deteriorate after 96 h of immersion in 0.5 M H_2_SO_4_. This indicates that K2 gradually loses their effectiveness over extended periods, leading to a degraded film that facilitates the penetration of aggressive sulfate ions (SO_4_^2−^) at the OL 37/SCG interface.

### 2.4. The Influence of Temperature

Potentiodynamic polarization analysis was performed between 298 K, 303 K, 313 K, 323 K, and 333 K to investigate how temperature impacts the anticorrosive performance of two spent coffee ground extracts (K1 and K2) at 1000 ppm on OL 37 steel in 1 M HCl and 0.5 M H_2_SO_4_ environments. The findings reveal a progressive rise in corrosion rates across both uninhibited and inhibited solutions as the temperature increases. Concurrently, a thermal rise triggers a decline in inhibition efficiency, pointing to a protective mechanism governed primarily by molecular adsorption onto the steel surface. This decline in performance at higher temperatures is driven by the enhanced desorption of the protective species. Finally, the relationship between temperature and the corrosion rate was modeled using both the Arrhenius and transition state equations.

$$i_{corr}=A\exp\left(\frac{-E_{a}}{RT}\right)$$

$$i_{corr}=\frac{RT}{Nh}\exp\left(\frac{\Delta S_{a}^{*}}{R}\right)\exp\left(\frac{\Delta H_{a}^{*}}{RT}\right)$$

**Figure 9:**
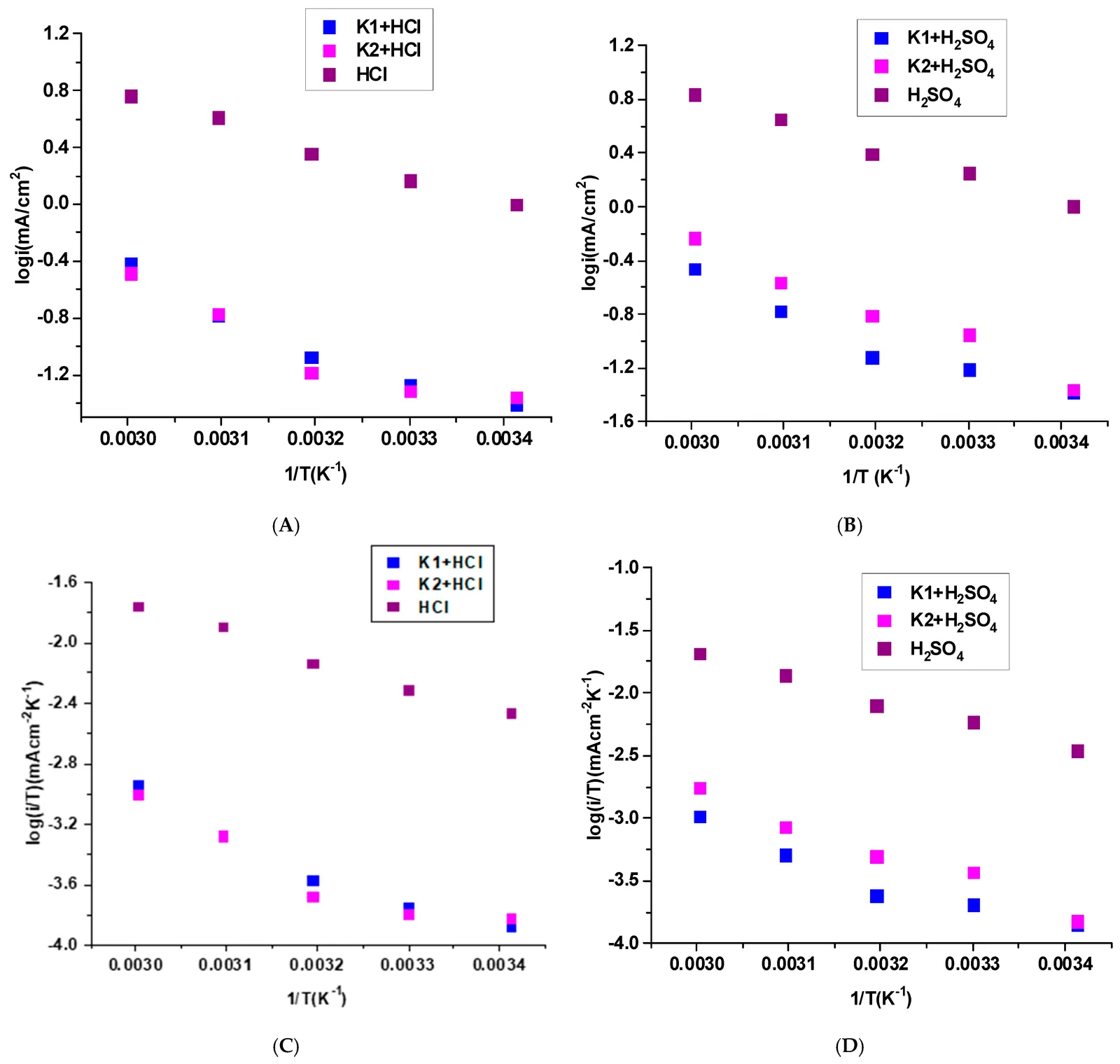
Arrhenius plots for OL 37 carbon steel in 1 M HCl (**A**,**C**) and 0.5 M H_2_SO_4_ (**B**,**D**) solutions in the absence and presence of 1000 ppm K1 and K2 green inhbitors at various temperatures (298 K, 303 K, 313 K, 323 K, and 333 K).

**Figure 10:**
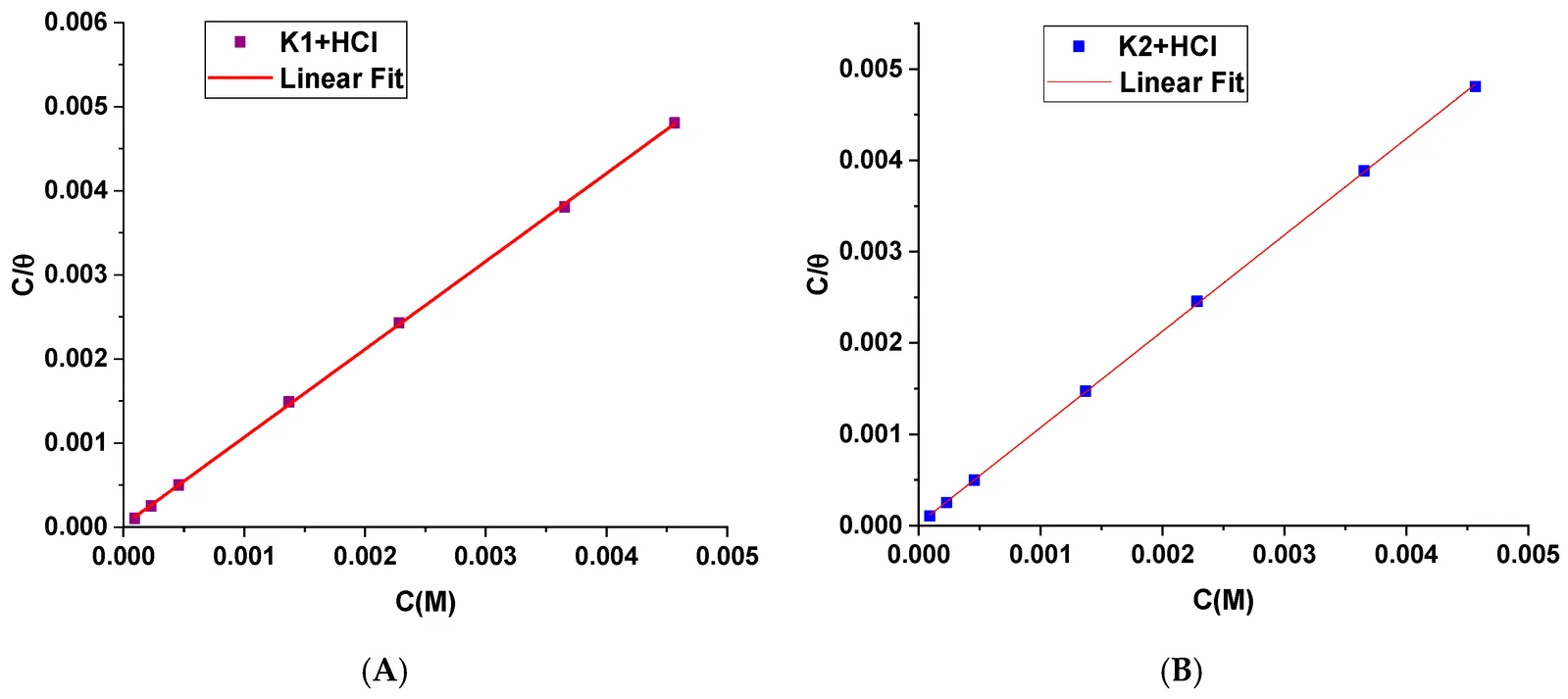

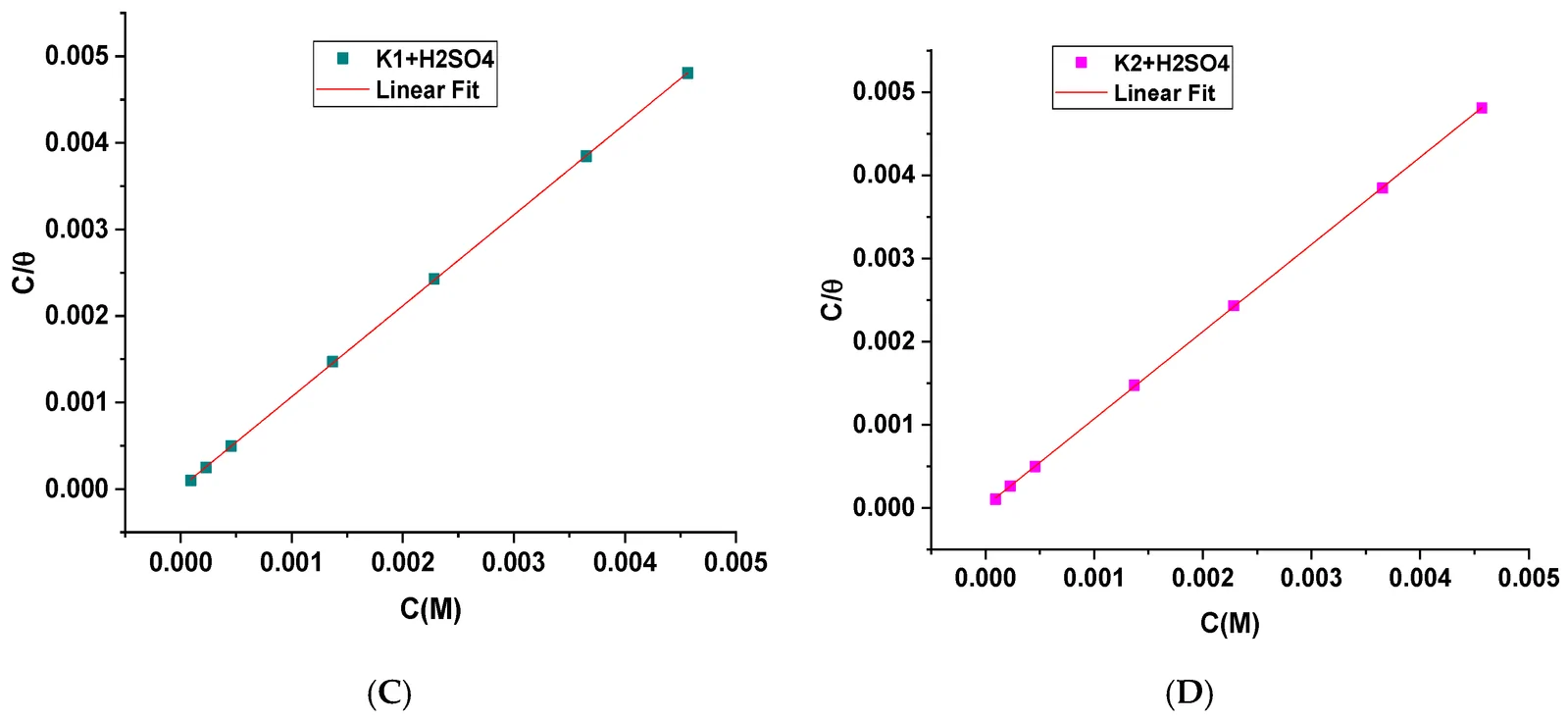
Langmuir adsorption isotherms for K1 and K2 green inhibitors on OL 37 carbon steel in 1 M HCl (**A**,**B**) and 0.5 M H_2_SO_4_ (**C**,**D**) solutions.

In these expressions, i_corr_ denotes the corrosion rate, A represents the pre-exponential factor, E_a_ signifies the apparent activation energy for the dissolution of OL 37 steel, T is the absolute temperature, and R is the universal gas constant. The terms ${∆H}_{a}^{*}$ and ${∆S}_{a}^{*}$ correspond to the apparent activation enthalpy and entropy, respectively, while h is Planck’s constant and N represents Avogadro’s number. The Arrhenius plots, showing log i_corr_ as a function of 1/T, are displayed in Figure 9A (for the 1 M HCl medium) and Figure 9B (for the 0.5 M H_2_SO_4_ medium), comparing the blank solutions with those containing the spent coffee ground (SCG) extracts. The E_a_ values were calculated directly from the slopes of these linear regressions and are compiled in Table 14. Furthermore, Figure 9C,D depicts the transition state plots of log i_corr_/T vs. 1/T. The resulting linear slopes to (−${∆H}_{a}^{*}$/R) and intercepts equal to ln(R/Nh) + (${∆H}_{a}^{*}$/R) were utilized to evaluate both ${∆H}_{a}^{*}$ and ${∆S}_{a}^{*}$, with the calculated thermodynamic parameters summarized in Table 14. As detailed in Table 14 and presented in Figure 10, the E_a_ values for the solutions containing the two spent coffee ground (SCG) extracts exceed those of the uninhibited media. This upward shift in activation energy highlights how temperature dictates the inhibition performance of these extracts. Figure 9A,B display the Arrhenius plots (log i_corr_ vs. T) for OL 37 steel in 1 M HCl and 0.5 M H_2_SO_4_, respectively, comparing the uninhibited and inhibited systems. The apparent activation energy (E_a_) parameters were calculated directly from the slopes of these linear regressions (−E_a_/R) and are summarized in Table 14. The transition state plots of log i_corr_/T vs. 1/T are depicted in Figure 9C,D. The resulting linear slopes −${∆H}_{a}^{*}$/R and intercepts equal to ln(R/Nh) + $({∆S}_{a}^{*}$/R) enabled the determination of the apparent activation enthalpy (${∆H}_{a}^{*})$ and entropy (${∆S}_{a}^{*}$) which are compiled in Table 14. Evaluation of these data shows that E_a_ values rise upon the addition of the spent coffee ground (SCG) extracts compared to the blank medium. This trend implies that the inhibitors establish a higher energy barrier for the corrosion process. This indicates that the adsorbed SCG film on the OL 37 steel surface effectively blocks the charge and mass transfer reactions, while confirming that the activation energy serves as a key indicator of how temperature impacts protection efficiency.

In the presence of the spent coffee ground (SCG) extracts, the concomitant rise in E_a_ and ${∆H}_{a}^{*}$, alongside negative ${∆S}_{a}^{*}$ values, demonstrates that the adsorption of phytochemical constituents alters the interfacial architecture. These green compounds interact with the metal surface via both electrostatic attraction and donor–acceptor mechanisms, establishing a protective adsorbed layer. This phenomenon decreases the density of active sites vulnerable to corrosion and induces a more ordered interfacial environment. Furthermore, the highly similar activation energies determined for K1 and K2 confirm that both extracts share a common adsorption pathway and possess equivalent thermal stability across the analyzed temperature range. The consistently positive ${∆H}_{a}^{*}$, values demonstrate the endothermic nature of the carbon steel dissolution process, confirming that the corrosion reaction is energy-dependent. In the inhibited systems, the negative ${∆S}_{a}^{*}$ values indicate that the activated complex generated during corrosion achieves a higher degree of structural order, which is attributed to the organized organic film at the interface. Consequently, the inhibition mechanism is predominantly driven by adsorption-induced surface shielding rather than by an elevation in the intrinsic electrochemical energy barrier.

### 2.5. Adsorption Isotherm

Adsorption isotherms provide significant insights into the interaction between organic substances and the sample surface. In this system, the enhanced efficiency of the SCG is driven by the adsorption process, which is generally considered the primary mechanism in the protective response on the OL 37 substrate. To evaluate how SCG concentration influences corrosion inhibition, the experimental data were fitted to an adsorption equilibrium model, specifically the Langmuir isotherm. This application demonstrated a strong correlation between the surface coverage of the substrate and the Langmuir equation. The adsorption mechanism was evaluated using the Langmuir isotherm model, expressed by the relationship:

θ/(1 − θ) = KC

where K represents the adsorption equilibrium constant, θ denotes the surface coverage of SCG on the substrate, and C is the SCG concentration. The surface coverage was determined using the following equation:

θ = (i_corr_ − i_inh_)/i_corr_

where i_corr_ and i_inh_ correspond to the corrosion current densities in the acidic solutions (1 M HCl, 0.5 M H_2_SO_4_) in the absence and presence of the inhibitor, respectively. The protective effect is ascribed to the adsorption of K1 and K2 onto the metal surface, a phenomenon strongly validated by correlation coefficients (R^2^) exceeding 0.99 (R^2^ = 0.9998 for K1 + HCl, 0.9999 for K2 + HCl, 0.9998 for K1 + H_2_SO_4_ and 0.9996 for K2 + H_2_SO_4_). This excellent linearity confirms that the Langmuir isotherm is highly applicable, demonstrating that the inhibition process is fundamentally governed by the adsorption of phytochemical constituents onto the substrate (Figure 10). The early-stage corrosion process of the metal substrate in 1 M HCl and 0.5 M H_2_SO_4_, along with the mitigation effect of SCG, occurs as follows:

$$Me+INH↔{Me\left(INH\right)}_{ads}↔{Me}^{n+}+ne^{-}+INH\;(Me=Fe,\;INH=K1,\;K2)$$

The adsorption of a substantial amount of SCG leads to the formation of a stable protective film on the OL 37 surface, effectively safeguarding (protecting) the metal from the corrosive medium. In this work, plotting the C_inh_/θ ratio against the inhibitor concentration (C_inh_) yielded straight lines with a slope of unity. The slope of this linear regression is a key parameter for evaluating the alignment of experimental data with the ideal Langmuir model (Figure 10). Theoretically, a slope of exactly 1, as noticed in this study (Table 15) validates the basic assumptions of the model: monolayer adsorption onto a homogeneous surface with negligible lateral interactions between the adsorbed species. Any deviation from unity typically signifies a departure from strict Langmuir behavior, often induced by surface heterogeneity, molecular interactions among the adsorbate species, or the occurrence of multilayer adsorption. Consequently, the slope provides essential insights into the validity of the Langmuir model and the nature of the underlying adsorption mechanism. This linear relationship confirms that the adsorption of SCG adheres to the Langmuir isotherm (Figure 10). The adsorption equilibrium constant (K_ads_) can be determined from the reciprocal of the intercept, and its calculated values are summarized in Table 15. High K_ads_ values clearly indicate efficient adsorption, signifying superior inhibition performance of the SCG on the OL 37 substrate in both 1 M HCl and 0.5 M H_2_SO_4_ media. This strong interaction influences the electrical characteristics of the double layer through its contact with the adsorbed phytochemicals, thereby enhancing the corrosion resistance of the metal. The correlation between the adsorption equilibrium constant (K_ads_) and the standard free energy of adsorption (ΔG^o^_ads_) is governed by the following equation:

$$\ln K_{ads}=-\left(\Delta G_{ads}^{o}/RT\right)$$

The linear plots of C_inh_/θ vs. C_inh_ yield slopes close to unity (1.044 for the K1 + HCl, 1.052 for the K2 + HCl, 1.050 for the K1 + H_2_SO_4_ and 1.048 for the K1 + H_2_SO_4_), confirming that the adsorption of these spent coffee ground-based inhibitors aligns with the Langmuir isotherm model. The negative values calculated for the standard free energy of adsorption ($∆G_{ads}^{{}^{\circ}})$ signify that the SCG adsorption onto the metal substrate is a spontaneous process. Furthermore, the magnitude of $∆G_{ads}^{{}^{\circ}}$ reflects an efficient interaction between the SCG biocomponents and the steel surface, providing direct insight into the nature of the binding mechanism. Generally, $∆G_{ads}^{{}^{\circ}}$ values around −20 kJ mol^−1^ or less negative indicate a process governed by electrostatic interactions (physisorption), whereas values approaching −40 kJ mol^−1^ or more negative suggest charge sharing or transfer between the OL 37 surface and the SCG molecules, characteristic of chemisorption (Table 15). In this study, the obtained data demonstrate that both physical and chemical adsorption mechanisms coexist simultaneously, with a predominant inclination toward chemisorption.

#### Mechanism of Inhibition

**Scheme 1:**
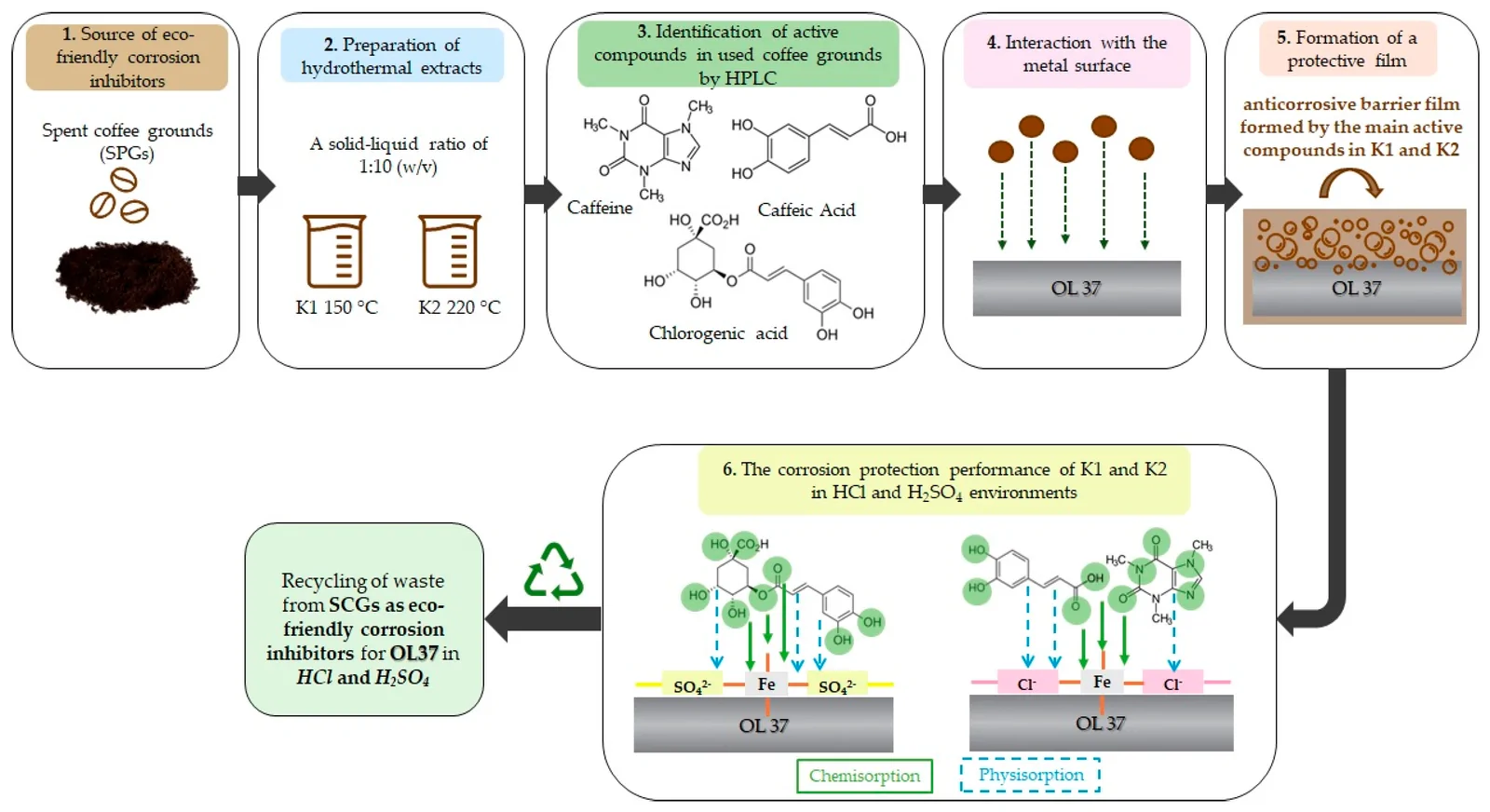
Proposed corrosion inhibition mechanism for SCG on OL 37 carbon steel in an aggressive acidic environment.

The inhibition mechanism was thoroughly evaluated, suggesting that both studied green inhibitors (K1 and K2) mitigate the corrosion of OL 37 steel in 1 M HCl and 0.5 M H_2_SO_4_ by the adsorption of their primary phytoconstituents at the substrate/electrolyte interface. This protective process is proposed to occur through a predominantly mixed physical and chemical adsorption mechanism, which appears to be governed by several key factors: the surface topography and charge of the substrate, the chemical architecture and ionic charge of the organic molecules, and the composition of the aggressive solution. The corrosion mitigation performance of these eco-friendly inhibitors seems to depend largely on the active surface coverage, molecular dimensions, and their specific binding modes with the metal surface. For these organic constituents, such as polyphenols, the presence of heteroatoms (oxygen) and functional groups (–OH, –COOH) provides highly active centers that are expected to facilitate the adsorption processes. The oxygen atoms exhibit the highest negative charge density, giving them a strong potential to bind onto the metal surface. Working in synergy, these phytoconstituents likely achieve corrosion protection through multiple adsorption modes. Physical adsorption may occur via electrostatic interactions, presumably mediated by the adsorption of electrolyte anions (Cl^−^ and SO_4_^2−^) onto the positively charged metal surface, which could facilitate the subsequent binding of the protonated organic species. Concurrently, chemical adsorption is assumed to take place through π donor–acceptor interactions, potentially involving the sharing of lone pair electrons from the oxygen donor atoms with the vacant d-orbitals of the surface iron (Fe) atoms. Collectively, these proposed adsorption pathways significantly reduce the active metal area exposed to the aggressive solution, thereby mitigating the degradation process. The proposed adsorption and inhibition mechanism for SCG on OL 37 carbon steel in 1 M HCl and 0.5 M H_2_SO_4_ media is illustrated in Scheme 1.

### 2.6. FT-IR Spectroscopic Characterization

**Figure 11:**
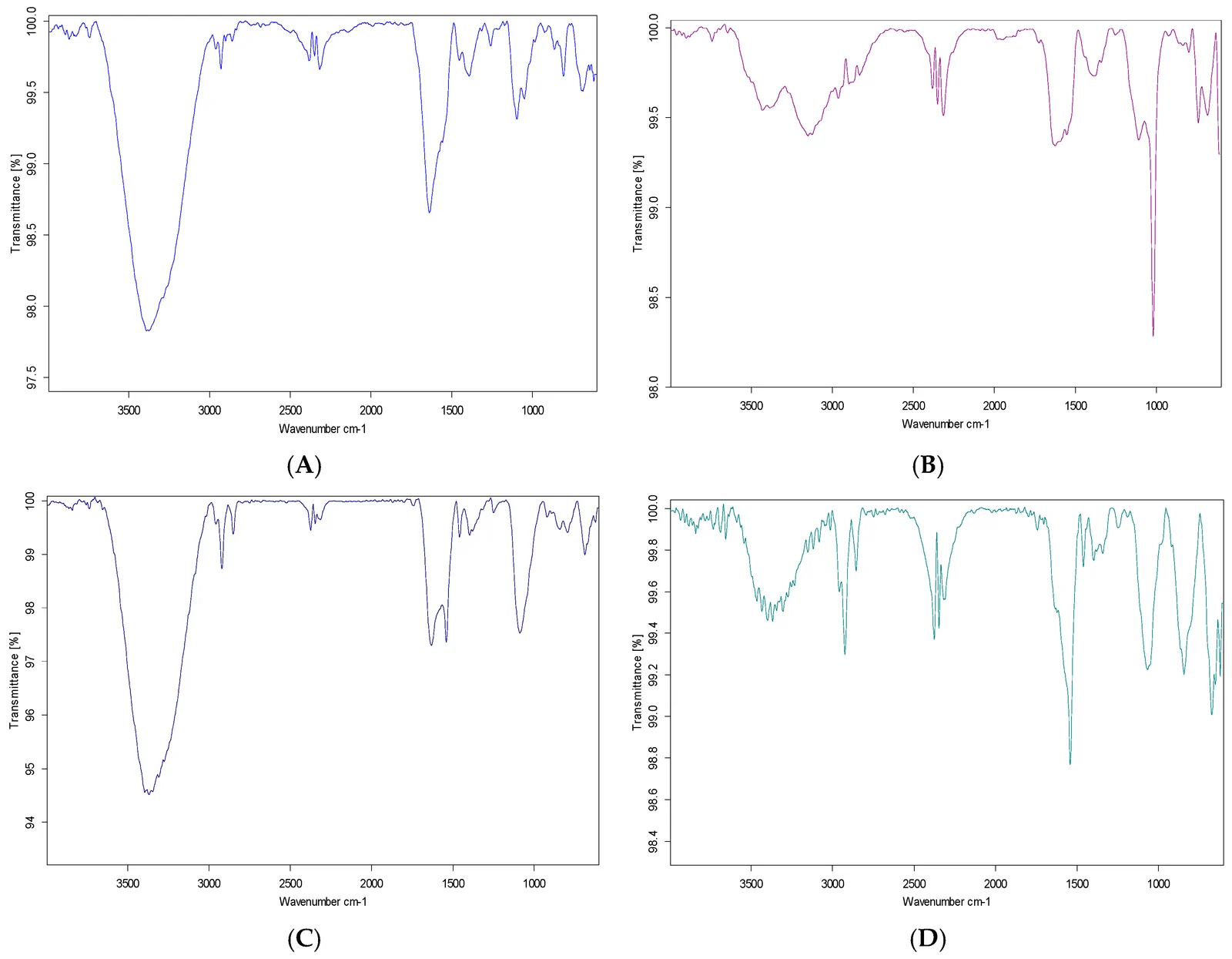
FT-IR spectra of the adsorbed film formed by K1 and K2 inhibitors on the OL 37 carbon steel surface after immersion in 1 M HCl (**A**,**B**) and 0.5 M H_2_SO_4_ (**C**,**D**).

Following their adsorption onto the OL 37 substrate during immersion in the aggressive medium, the characteristic absorption bands of the K1 and K2 green inhibitors were identified via FT-IR analysis. As illustrated in Figure 11, the FT-IR spectra were evaluated to characterize the protective layer formed on the OL 37 surface and to gain further insight into the binding interactions at the metal/solution interface. The spectra recorded for the steel samples after exposure to K1 and K2 are indicative of an adsorbed organic film. As shown in Figure 11A,B, several prominent absorption bands confirm the presence of organic constituents originating from the spent coffee ground (SCG) extract. Significantly, the majority of these peaks mirror those found in the SCG extract spectrum, confirming that they stem from the phytochemical components of the inhibitor and are directly associated with their attachment to the steel surface. Specifically, the broad band in the 3400–3300 cm^−1^ region is assigned to the O-H stretching vibrations of hydroxyl groups from phenolic compounds and/or co-adsorbed water molecules. Additionally, the bands located at approximately 2950 and 2850 cm^−1^ correspond to the asymmetric and symmetric C-H stretching vibrations -CH_2_ and -CH_3_ groups, demonstrating the presence of aliphatic fragments derived from the organic matrix of the extract. In the 1650–1600 cm^−1^ domain, the detected band is primarily ascribed to the bending vibration of adsorbed water (H-O-H), though minor contributions from surface iron oxide species cannot be entirely ruled out. Additional peaks in the 1480–1350 cm^−1^ range are assigned to the deformation vibrations of aliphatic groups, further verifying the deposition of organic material onto the steel surface. The intense band spanning the 1250–1100 cm^−1^ region is characteristic of C–O stretching vibrations stemming from phenolic, alcoholic, and ether moieties, while the signals between 1050 and 1000 cm^−1^ are similarly associated with C–O vibrations typical of the spent coffee ground (SCG) compounds.

Subtle shifts in peak positions, intensity variations, and band broadening compared to the pristine extract (Figure 1) suggest potential interactions between the oxygen-containing functional groups and the metal substrate. Nevertheless, the preservation of specific absorption bands characteristic of the spent coffee ground (SCG) phytoconstituents on the treated OL 37 samples provides definitive evidence of inhibitor adsorption. Therefore, these FT-IR data should be understood not as a standalone proof of novel chemical bond formation, but rather as a clear confirmation that the phytochemical constituents have anchored to the steel surface. Overall, the spectroscopic findings support the development of a protective organic film composed of adsorbed SCG compounds, which acts as a physical barrier that restricts direct contact between the aggressive medium and the metal substrate, thereby driving the corrosion inhibition process.

### 2.7. SEM and EDX Examination

**Figure 12:**
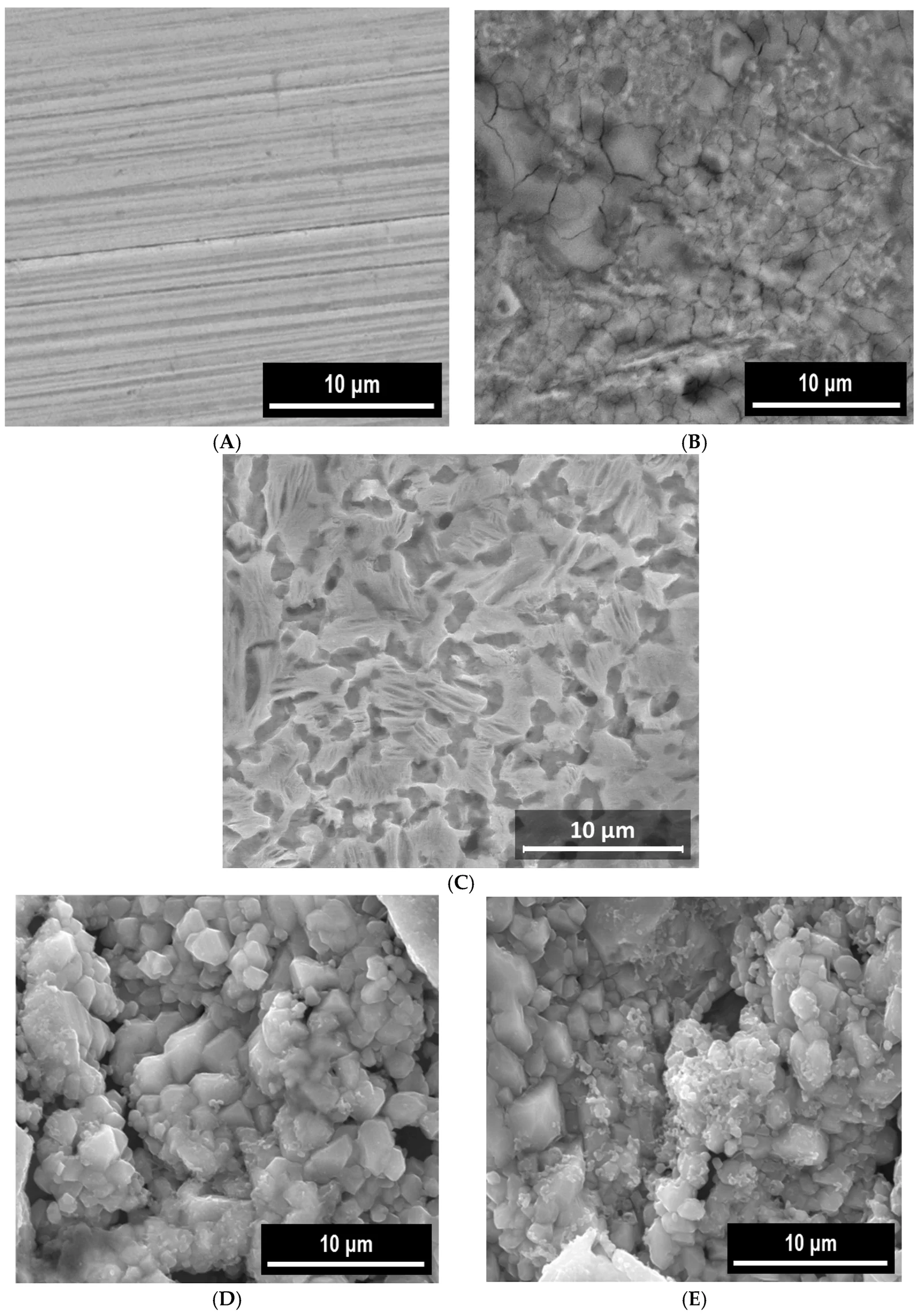

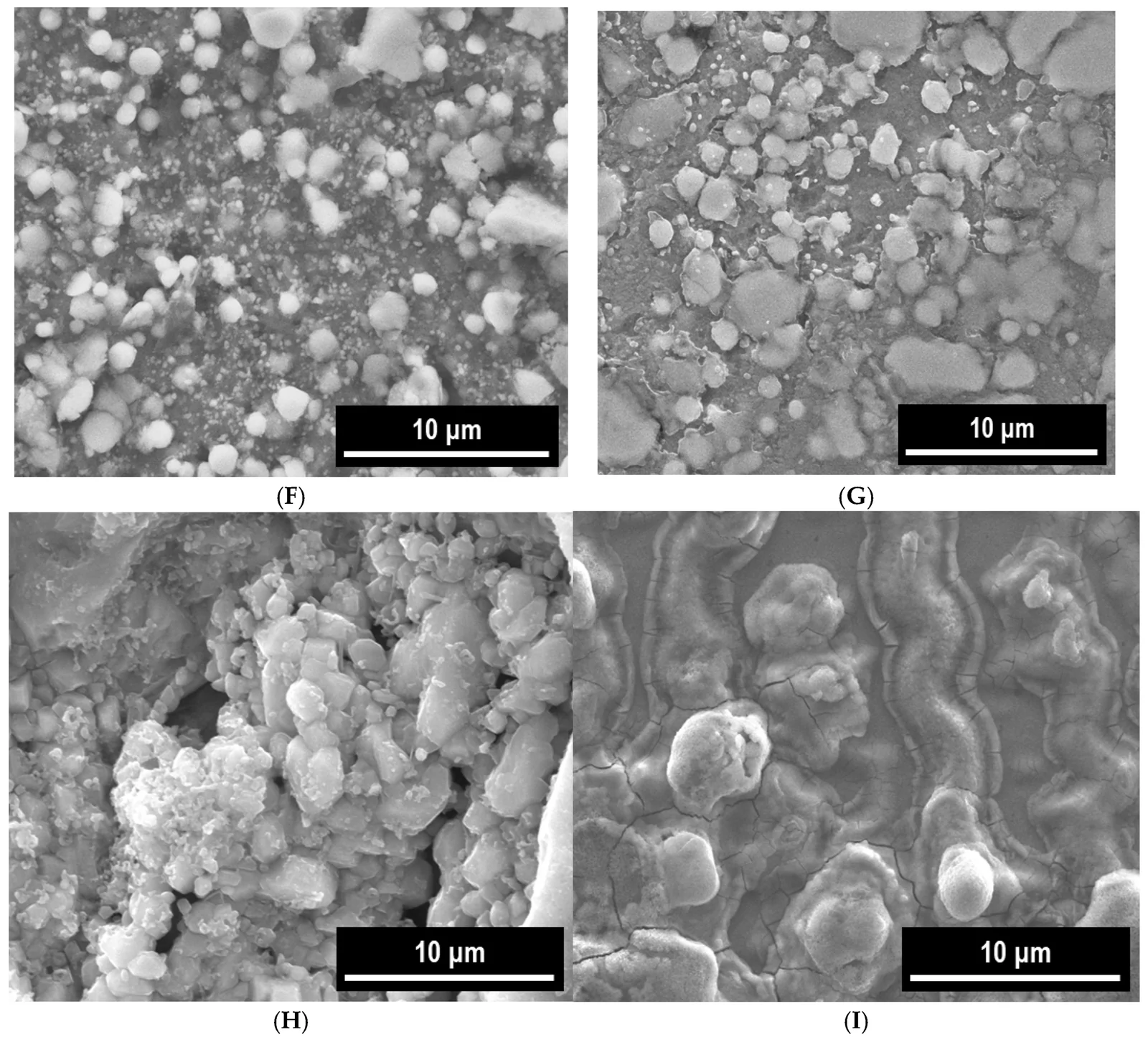
SEM morphology of carbon steel surface: (**A**) polished carbon steel (**B**) OL 37 + HCl, (**C**) OL 37 + H_2_SO_4_, (**D**) 1000 ppm K1 + HCl, (**E**) 1000 ppm K2 + HCl_,_ (**F**) 1000 ppm K1 + H_2_SO_4_, (**G**) 1000 ppm K2+ H_2_SO_4_, (**H**) 1000 ppm K1 + HCl 1000 ppm and (**I**) K2+ H_2_SO_4_ after 96 h, immersion time.

Figure 12 displays the SEM micrographs of the OL 37 carbon steel surface after a 4 h immersion in 1 M HCl and 0.5 M H_2_SO_4_ solutions, both in the absence and presence of 1000 ppm eco-friendly inhibitors extracted from spent coffee grounds (K1 and K2). Figure 12A shows an SEM micrograph of the freshly polished carbon steel surface. As shown in Figure 12B,C, the OL 37 substrate suffered severe surface degradation when immersed in the uninhibited 1 M HCl and 0.5 M H_2_SO_4_ environments. Conversely, Figure 12C–F reveal that the addition of 1000 ppm SCG extracts (K1 and K2) significantly improved the surface morphology compared to the unprotected sample. This enhancement demonstrates the remarkable corrosion protection capability of these green inhibitors, which form an adsorbed protective film on the OL 37 surface, limiting its contact with the aggressive media and effectively mitigating corrosion.

**Figure 13:**
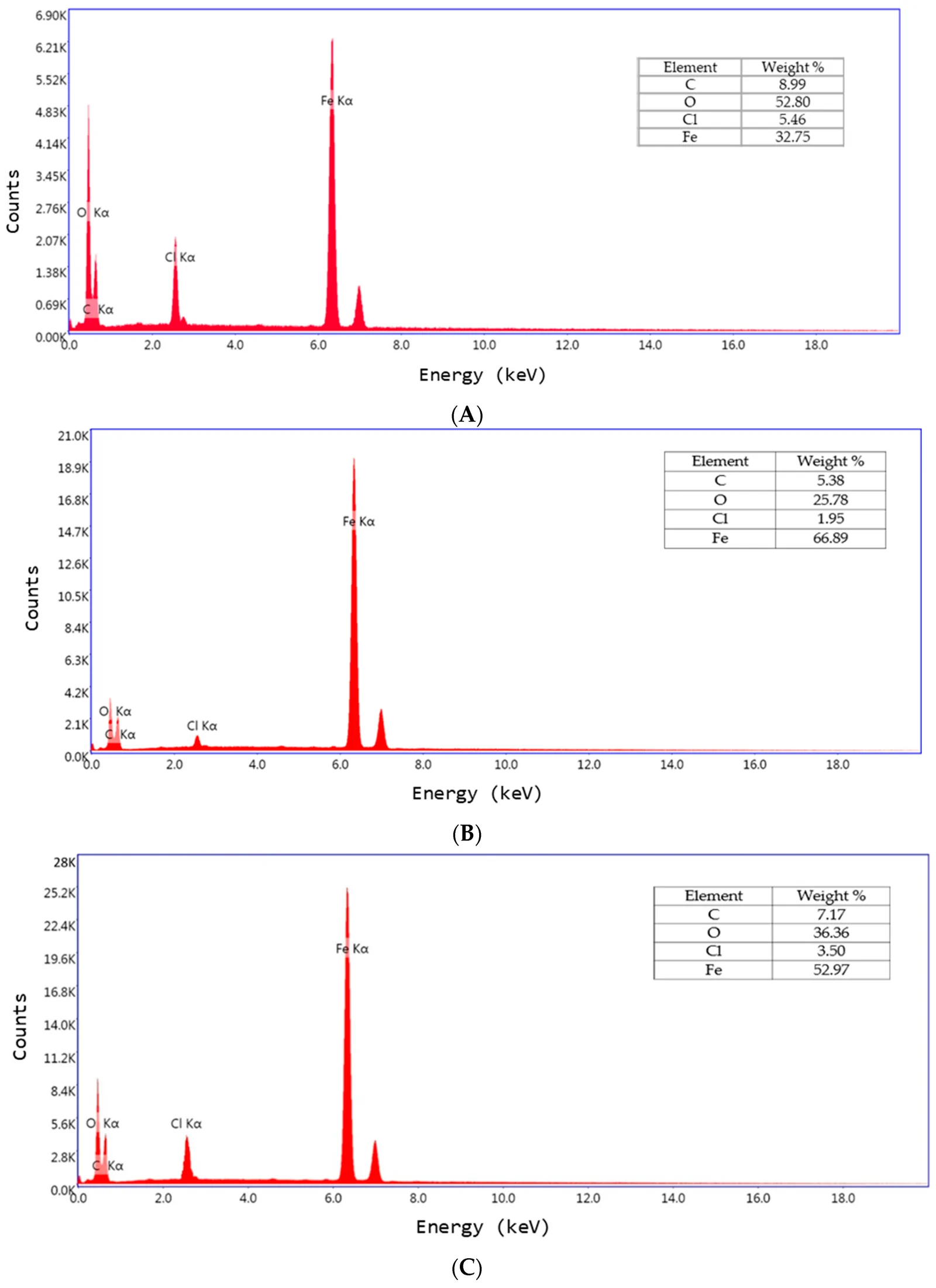

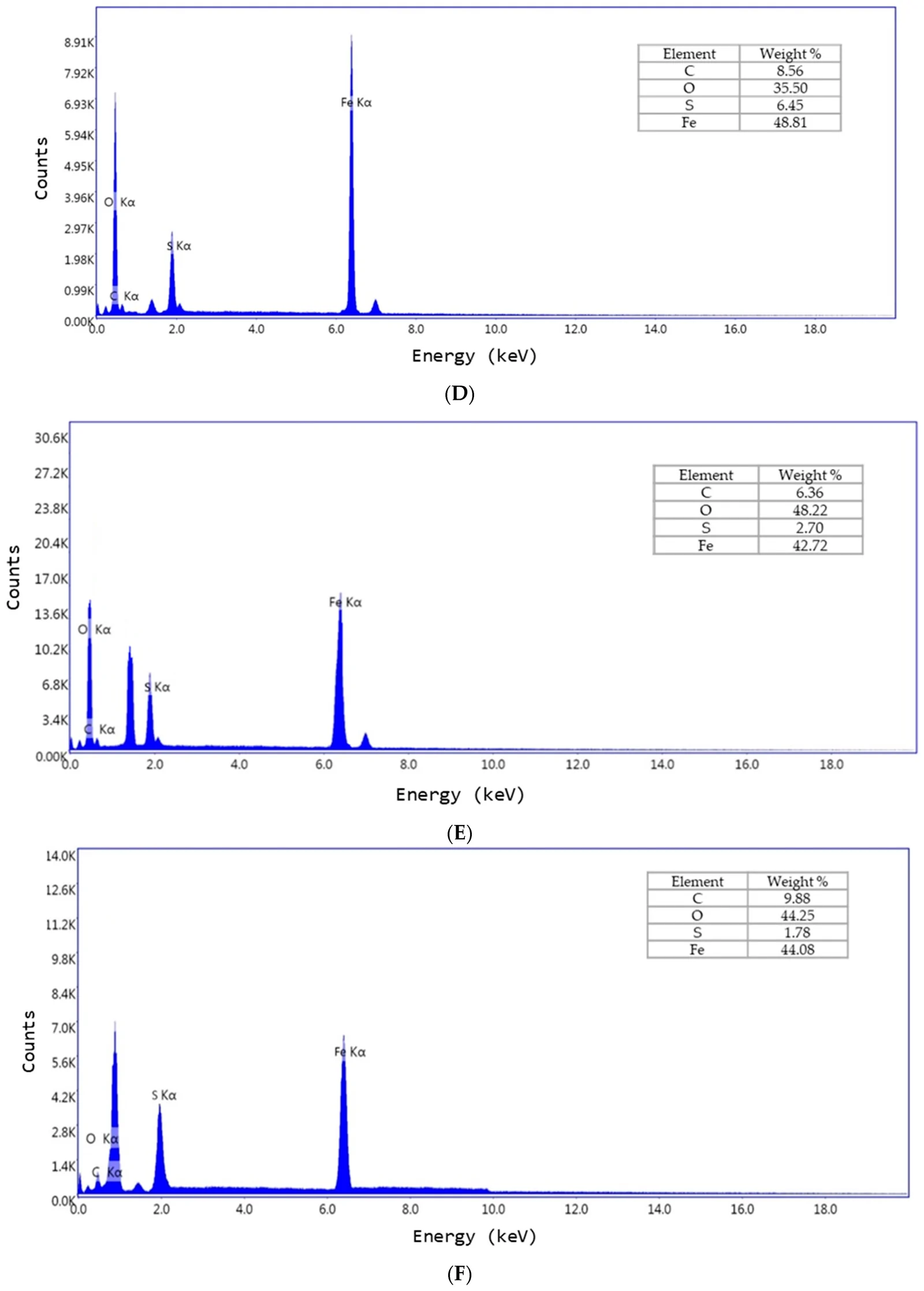
EDS spectra obtained for (**A**) OL 37 + HCl, (**B**) OL 37 + H_2_SO_4_, (**C**) 1000 ppm K1 + HCl, (**D**) 1000 ppm K2 + HCl, (**E**) 1000 ppm K1 + H_2_SO_4_ and (**F**) 1000 ppm K2 + H_2_SO_4_.

Figure 13 presents the EDS spectra obtained for the carbon steel substrates in both the uninhibited and inhibited (K1 and K2) conditions. For the uninhibited OL 37 carbon steel, immersion in HCl (Figure 13A) resulted in intense chlorine (Cl) and oxygen (O) peaks, while immersion in H_2_SO_4_ (Figure 13B) produced prominent sulfur (S) and oxygen (O) signals, confirming the accumulation of corrosion products on the metal surface. Conversely, the EDS spectra for the inhibited OL 37 samples (Figure 13C–E) display peaks for C, O, Fe, S, and Cl, which indicates the formation of an adsorbed protective film on the substrate. Notably, the intensities of these aggressive elements are significantly reduced compared to the unprotected samples, demonstrating effective surface protection.

While elevated concentrations of Cl, S, and O are indicative of accelerated corrosion in the uninhibited hydrochloric and sulfuric acid solutions, the presence of the spent coffee ground (SCG) extracts (K1 and K2) markedly suppresses these elemental signals. This decrease points to the adsorption of inhibitor molecules at the metal/solution interface, effectively isolating the steel from the corrosive medium. The reduction in these elemental intensities confirms that iron dissolution and aggressive ion attacks were successfully mitigated. These results strongly correlate with the FTIR results, further validating the formation of a stable protective film on the steel substrate.

## 3. Materials and Methods

### 3.1. Materials

The used coffee grounds used in this study were collected from the daily consumption of whole bean coffee by regular users, using an automatic coffee machine equipped with a built-in grinder. It resulted from the preparation of Lavazza brand commercial coffee, the Crema e Aroma blend, composed of 60% Arabica coffee from the São Paulo region (Brazil, natural processing) and Honduras (washed processing) and 40% Robusta coffee grown in Uganda. In this study, corrosion tests were performed on an OL 37 working electrode, with the following composition.: C% 0.15, Si 0.09%, Mn 0.4%, Fe% 99.293, P% 0.023, S% 0.02, Al% 0.022, Ni% 0.001, Cr% 0.001.

The corrosive environments were:

- 1 M HCl, achieved by diluting 36% HCl, analytical grade (Merck, Rahway, NJ, USA) with bi-distilled water;
- 0.5 M H_2_SO_4_, obtained by diluting 96% H_2_SO_4,_ analytical grade (Merck, Rahway, NJ, USA) with bi-distilled water.

### 3.2. Methods

#### 3.2.1. Preparation of Used Coffee Grounds Extracts

**Figure 14:**
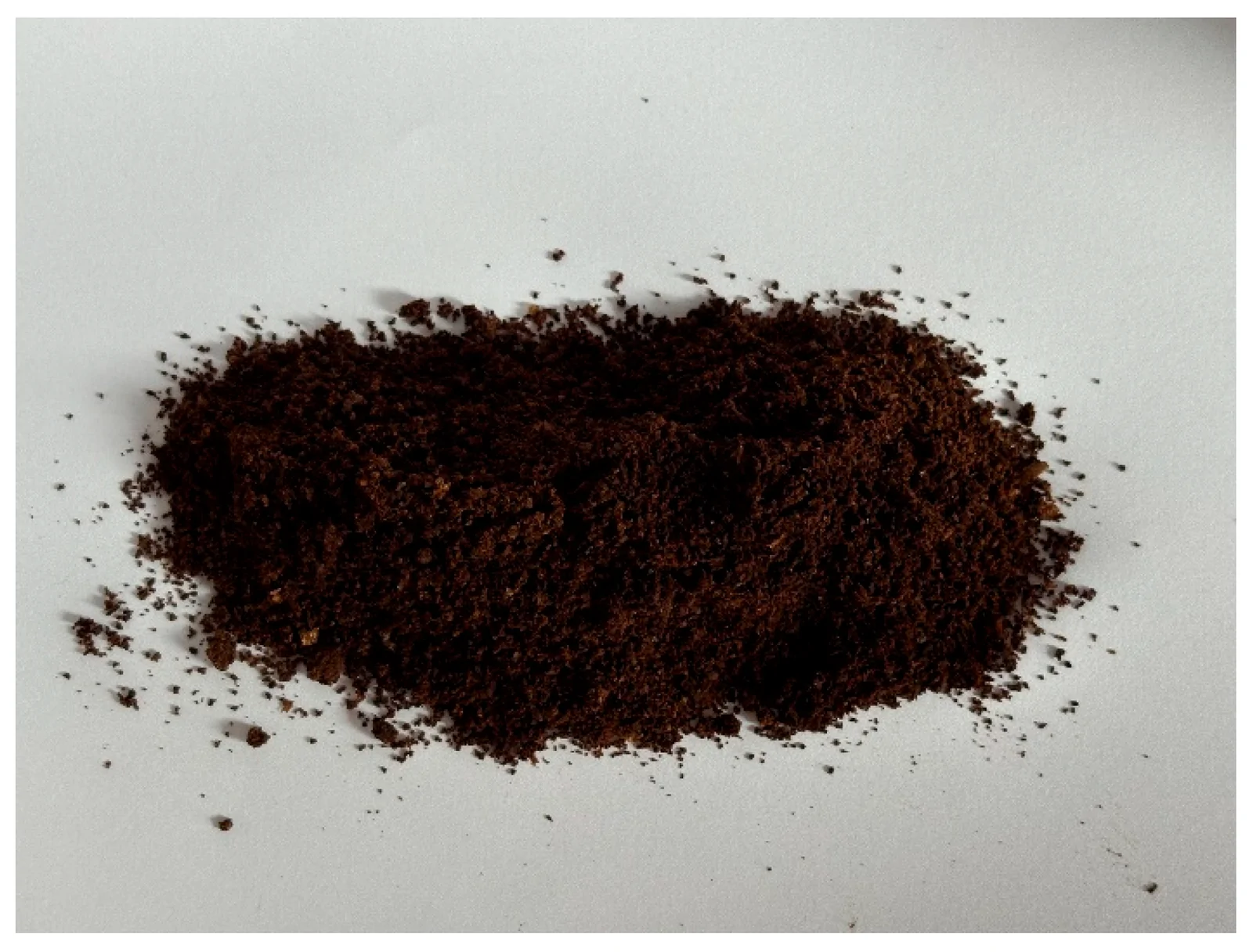
Spent Coffee Grounds powder.

A pretreatment procedure was implemented to remove impurities and standardize the material used. The spent coffee grounds were dried in a Memmert oven at 25 °C until a constant weight was reached, then stored in a desiccator to prevent rehydration caused by the absorption of water vapor from the atmosphere (Figure 14).

**Scheme 2:**
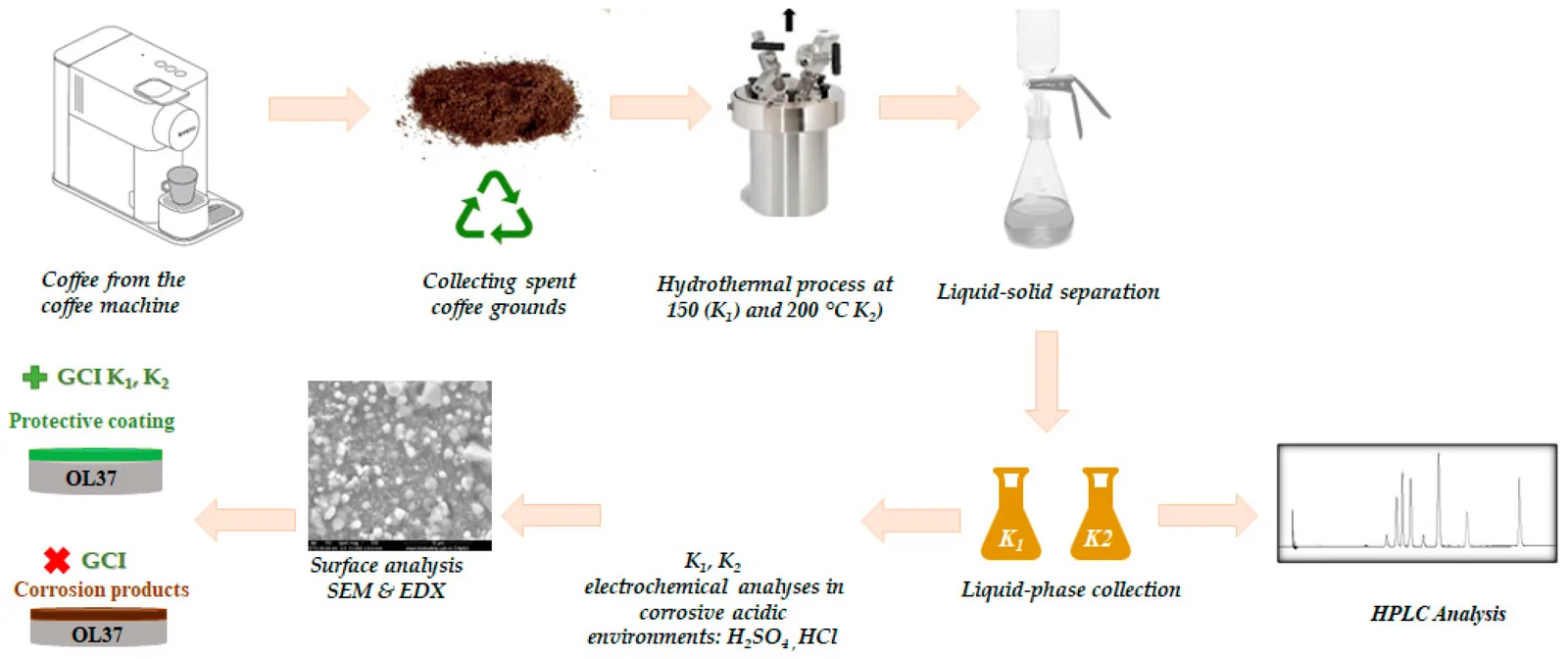
Diagram of the extraction, characterization, and utilization of SCG for OL 37 green corrosion protection.

The corrosion inhibitors were obtained from spent coffee grounds via hydrothermal extraction of bioactive compounds. The process was conducted for 60 min, with 300 rpm at varying temperatures (150 °C and 220 °C), and the pressure is self-generated (Scheme 2). A solid-to-liquid ratio of 1:10 (*w*/*v*), was used, more precisely 30 g spent coffee grounds/300 mL ultrapure double-distilled water. The hydrothermal extraction process was carried out using the FCF-1 autoclave, manufactured by ZZKD 307 (Zhengzhou, China).

Following the hydrothermal extraction of spent coffee grounds at 150 °C and 220 °C, three distinct phases were obtained (Scheme 2).

- The gaseous phase, observed upon opening the autoclave, consisted mainly of low-molecular-weight compounds such as carbon monoxide (CO), carbon dioxide (CO_2_), and methane (CH_4_).
- The liquid phase was separated from the solid residue by vacuum filtration using a Büchner funnel and a vacuum pump. These analyses aimed to evaluate the potential of the extracts as green corrosion inhibitors and to determine the composition of the main extracted compounds.
- The solid phase was not the focus of the present study. However, it was washed with distilled water until clear solutions free of visible impurities were obtained. Subsequently, the solid residues were dried in an oven and stored for potential future applications (Scheme 2).

**Figure 15:**
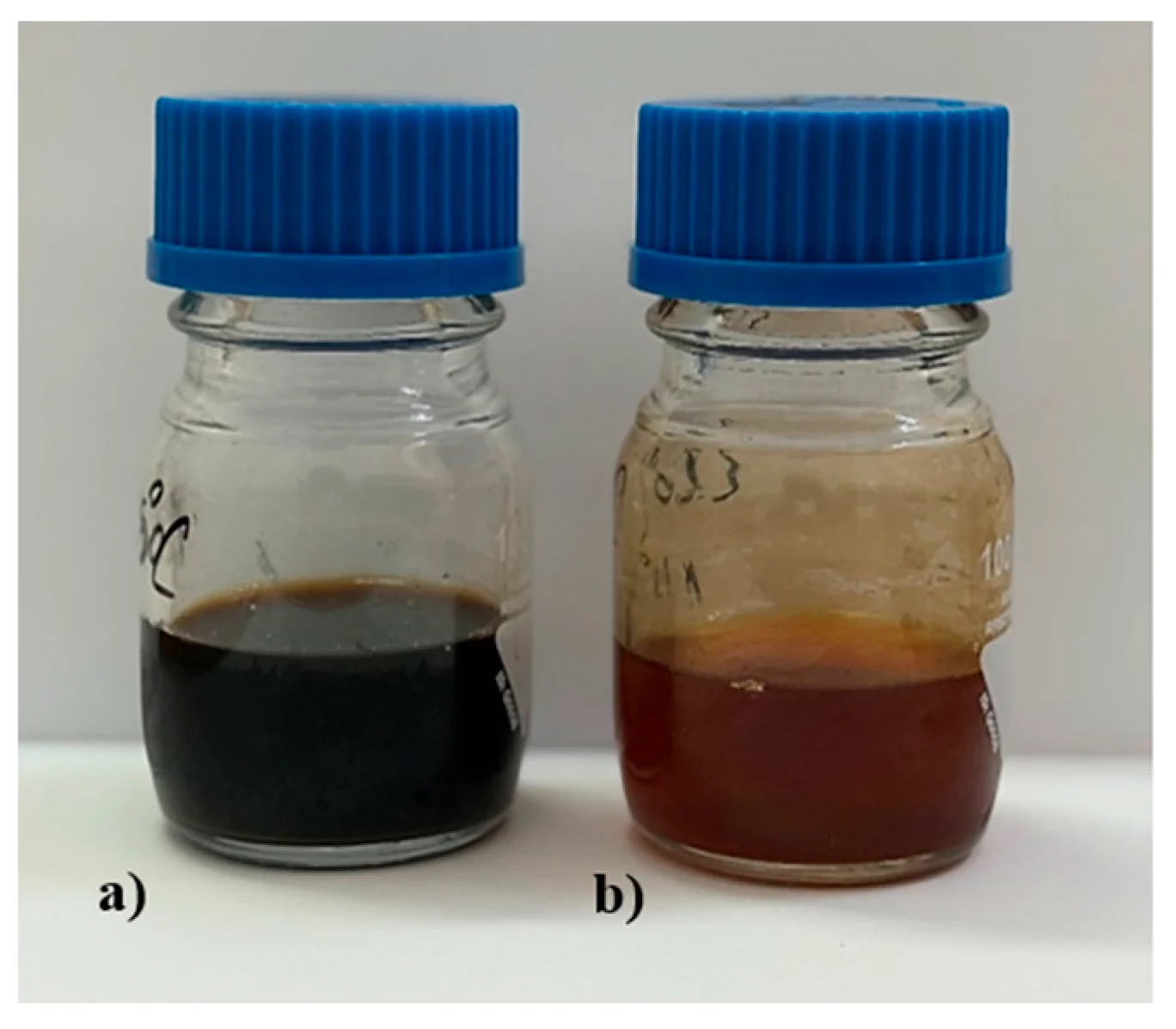
Coffee extracts obtained from the hydrothermal process, (a) K_1_, at 150 °C and (b) K_2_, at 220 °C.

The extract from 150 °C, was noted as K1, and the one from 220 °C, K2 (Figure 15).

#### 3.2.2. Characterization of Dry-Powder SCG

Using a Bruker Optics Tensor 37 spectrometer (with ATR), Ettlingen, Germany, a Fourier-transform infrared spectroscopy (FTIR) analysis was performed.

#### 3.2.3. Characterization of Aqueous Extracts by HPLC Analysis

Used coffee grounds were dried, ground, and sieved before analysis. For extraction, 0.500 g of dry sample was mixed with 10.0 mL of methanol/water (50:50, *v*/*v*), acidified with 0.1% formic acid, then the mixture was subjected to ultra-sonication for 30 min at room temperature and centrifuged for 10 min. The supernatant was filtered thru a 0.22 µm membrane before injection into the HPLC system.

The chromatographic analysis of spent coffee grounds extract was performed using an HPLC system equipped with a UV–Vis detector (Waters 2489), Milford, Massachusetts. Separation was carried out on a C18 column (250 × 4.6 mm, 5 µm) maintained at 30 °C. The mobile phase consisted of 0.1% trifluoroacetic acid in water (A) and 0.1% trifluoroacetic acid in acetonitrile (B), using gradient elution at a flow rate of 1.0 mL min^−1^. The gradient program was set as follows: 0–5 min, 5% B; 5–20 min, 5–15% B; 20–35 min, 15–25% B; 35–40 min, 25–35% B, followed by column re-equilibration to the initial conditions. The injection volume was 10 µL.

Chromatographic detection was carried out at 280 nm. Standard solutions of caffeine, gallic acid, (+)-catechin, caffeic acid, chlorogenic acid, syringic acid, (−)-epicatechin, p-coumaric acid, ferulic acid, and ellagic acid were prepared at 1000 µg mL^−1^ and used for compound identification and quantification. Calibration curves were established in the concentration range of 10–400 µg mL^−1^. The compounds were identified by comparing their retention times with those of the corresponding standards and quantified using external calibration.

#### 3.2.4. Electrochemical Measurements

In this study, corrosion experiments were performed on an OL 37 carbon steel substrate and the corrosive media consisted of 0.5 M H_2_SO_4_ and 1 M HCl solutions. Cylindrical OL 37 carbon steel specimens, featuring an active surface area of 0.5 cm^2^, were utilized to provide a consistent and well-defined exposure zone. The samples (carbon steel) underwent mechanical polishing using a series of SiC abrasive papers (600 to 4000 grit) to achieve a mirror-like finish. To eliminate organic contaminants, the specimens were degreased with acetone, followed by a thorough rinse with double-distilled water. After drying at ambient temperature, the prepared working electrodes were integrated into the electrochemical cell.

The corrosion inhibition performance of carbon steel was evaluated using a VoltaLab-PGZ 402 potentiostat/galvanostat system, controlled by Voltamaster 4 software. The study employed a comprehensive suite of electrochemical techniques, specifically potentiostatic polarization, potentiodynamic polarization, and electrochemical impedance spectroscopy (EIS). These measurements were conducted within a standard three-electrode cell arrangement, where OL 37 carbon steel cylindrical specimen functioned as the working electrodes (WEs). A saturated calomel electrode (SCE) served as the reference, while a platinum plate was utilized as the auxiliar electrode. All experimental procedures were carried out at a constant temperature of 25 °C under naturally aerated, quiescent conditions, strictly avoiding any mechanical stirring or agitation. To ensure reproducibility, all tests were performed in triplicate (*n* = 3), with the resulting data reported as mean values accompanied by the standard deviation (±SD). Corrosion assessments of OL 37 carbon steel were carried out in 0.5 M H_2_SO_4_ and 1 M HCl solutions, both in the absence and presence of two distinct green inhibitors derived from spent coffee grounds (SCGs), designated as K1 and K2. The electrochemical behavior was investigated using potentiodynamic polarization and electrochemical impedance spectroscopy (EIS). Tafel polarization curves were recorded by scanning the potential within (±250 mV) vs. OCP at a scan rate of 2 mV s^−1^. Key corrosion parameters, including corrosion current density (i_corr_) and Tafel slopes (b_a_ and b_c_), were determined by extrapolating the linear branches to the corrosion potential (E_corr_). EIS measurements were performed at the steady-state potential at OCP, covering a frequency range from 100 kHz to 40 mHz at a resolution of 10 points per decade. To ensure reproducibility and statistical consistency, all experiments were performed in triplicate.

#### 3.2.5. Surface Charaterization

Surface analysis and characterization was conducted to evaluate the morphological and chemical modifications of the OL 37 carbon steel substrate with and without green inhibitors. Micrographs were acquired using a Quanta Inspect F50 (FEI Company, Hillsboro, OR, USA) Scanning Electron Microscope (SEM), equipped with a Field Emission Gun (FEG) that ensures a high spatial resolution of 1.2 nm. To assess the elemental distribution and the formation of protective layers, the system was coupled with an Energy Dispersive X-ray (EDX) spectrometer (Mahwah, NJ, USA), providing an MnK resolution of 133 eV Kα. FTIR analysis was performed to identify the functional groups of the inhibitors adsorbed on the steel surface. These measurements were carried out using a Bruker Optics Tensor 37 spectrometer (Ettlingen, Germany) in ATR mode, covering a spectral range of 4000–650 cm^−1^ at a resolution of 4 cm^−1^.

## 4. Conclusions

This study highlights the remarkable performance of spent coffee ground (SCG) extracts (K1 and K2) as sustainable, eco-friendly corrosion inhibitors for OL 37 carbon steel in 1 M HCl and 0.5 M H_2_SO_4_ solutions. Electrochemical assessments, utilizing potentiodynamic polarization and electrochemical impedance spectroscopy, revealed a significant reduction in corrosion rates upon the addition of both extracts. Maximum inhibition efficiencies reached approximately 96% and 95% for K1 and K2 in 1 M HCl, and 95% for both extracts in 0.5 M H_2_SO_4_ at concentrations of 800 ppm and 1000 ppm. Advanced surface characterization (SEM, EDX, FT-IR) verified the development of a protective film, offering explicit evidence of the inhibitors’ capability to shield the steel and mitigate aggressive corrosive attacks.

The electrochemical data confirmed that these SCG-derived inhibitors operate by a mixed-type mechanism, simultaneously suppressing anodic metal dissolution and cathodic hydrogen evolution. This mitigation is driven by the adsorption of active phytochemical constituents onto the steel surface, establishing a protective barrier. The adsorption process conforms to the Langmuir isotherm model, while the negative ΔG°_ads_ values denote a spontaneous process governed by comprehensive physisorption and chemisorption interactions.

The addition of K1 and K2 in hydrochloric acid provides enhanced, long-term protection compared to their performance in sulfuric acid.

HPLC phytochemical profiling unveiled a complex matrix rich in phenolic compounds, prominently featuring gallic, caffeic, syringic, p-coumaric, ellagic, and ferulic acids. The abundance of electron-donating functional groups within these molecules facilitates their anchoring at the metal/solution interface, reinforcing the protective barrier through mixed physical and chemical interactions.

In conclusion, this research establishes SCG extracts (K1 and K2) as highly promising, green alternatives to traditional synthetic corrosion inhibitors in acidic media. Future investigations will employ theoretical modeling to resolve the molecular-level interactions, alongside performance testing under harsher conditions and within diverse corrosive environments.

## Figures and Tables

**Table 1:** Main compounds present in the SCGs extracts.

No.	Identified Compound	Retention Time (min)	Signal (mAU) K_1_	Content (mg L^−1^) K_1_	Signal (mAU) K_2_	Content (mg L^−1^) K_2_	Chemical Structure
1.	Caffeine	2.590	67.17	4.45	90.13	5.57	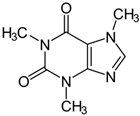
2.	Gallic Acid	3.617	256.87	1.09	320.48	2.04	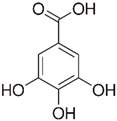
3.	(+) Catechin	13.987	67.17	1.31	25.54	0.55	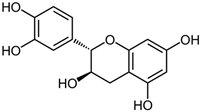
4.	Caffeic Acid	15.193	252.44	5.94	350.60	8.15	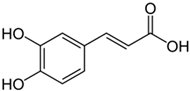
5.	Chlorogenic acid	15.903	393.06	5.47	125.53	1.3	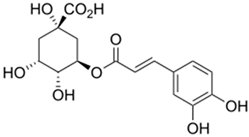
6.	Syringic acid	16.893	366.99	25.35	470.05	30.15	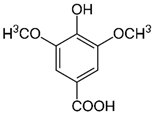
7.	(−)Epicatechin	18.447	60.65	0.48	15.35	0.13	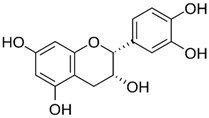
8.	p-Coumaric acid	20.287	500.60	0.78	550.85	1.2	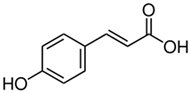
9.	Ferulic acid	23.733	184.58	1.82	250.42	2.55	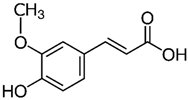
10.	Ellagic acid	30.043	373.51	1.29	130.53	0.51	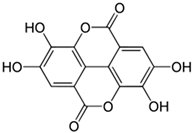

**Table 2:** Kinetic parameters of OL 37 in 1 M HCl at various concentrations of K1 at temperature of 25 °C.

Concentration (ppm)	i_corr_ (mA cm^−2^)	R_p_ Ω cm^−2^	R_mpy_	P_mm/year_	K_g_ g m^−2^ h^−1^	E (%)	-E_corr_ (mV)	b_a_ (mV dec^−1^)	-b_c_ (mV dec^−1^)	θ	-OCP (mV)
0	0.855 ± 0.020	20	399	10.12	9	-	531 ± 1	86	113	-	535
20	0.087 ± 0.001	133	40.69	1.03	0.918	89	472 ± 2	60	80	0.89	489
50	0.081 ± 0.002	156	37.98	0.96	0.85	90	459 ± 2	58	91	0.90	465
100	0.074 ± 0.002	180	34.53	0.87	0.76	91	426 ± 3	67	90	0.91	436
300	0.063 ± 0.001	165	29.4	0.74	0.63	92	447 ± 3	68	82	0.92	451
500	0.047 ± 0.003	244	21.93	0.55	0.45	94	479 ± 1	70	90	0.94	481
800	0.037 ± 0.002	464	12.6	0.32	0.28	96	457 ± 2	63	92	0.96	465
1000	0.039 ± 0.001	324	18.2	0.46	0.41	95	478 ± 1	76	84	0.95	480

**Table 3:** Kinetic parameters of OL 37 in 1 M HCl at various concentrations of K2 at temperature of 25 °C.

Concentration (ppm)	i_corr_ (mA cm^−2^)	R_p_ Ω cm^−2^	R_mpy_	P_mm/year_	K_g_ g m^−2^ h^−1^	E (%)	-E_corr_ (mV)	b_a_ (mV dec^−1^)	-b_c_ (mV dec^−1^)	θ	-OCP (mV)
0	0.855 ± 0.020	20	399	10.12	9	-	531 ± 1	86	113	-	535
20	0.100 ± 0.001	129	49.46	1.25	1.11	87	473 ± 2	59	62	0.87	486
50	0.073 ± 0.002	248	34.06	0.86	0.77	91	486 ± 1	76	80	0.91	491
100	0.062 ± 0.002	216	29.4	0.74	0.66	92	475 ± 2	65	106	0.92	485
300	0.056 ± 0.003	229	26.13	0.66	0.59	93	473 ± 2	64	88	0.93	479
500	0.057 ± 0.001	271	26.6	0.67	0.60	93	473 ± 1	68	86	0.93	479
800	0.050 ± 0.002	292	23.3	0.59	0.52	94	471 ± 2	61	85	0.94	477
1000	0.042 ± 0.003	299	20.53	0.52	0.46	95	422 ± 3	62	95	0.95	438

**Table 4:** Kinetic parameters of OL 37 in 0.5 M H_2_SO_4_ at various concentrations of K1 at temperature of 25 °C.

Concentration (ppm)	i_corr_ (mA cm^−2^)	R_p_ Ω cm^−2^	R_mpy_	P_mm/year_	K_g_ g m^−2^ h^−1^	E (%)	-E_corr_ (mV)	b_a_ (mV dec^−1^)	-b_c_ (mV dec^−1^)	θ	-OCP (mV)
0	0.898 ± 0.020	16	419	10.63	9.45	-	491 ± 1	97	94	-	495
20	0.081 ± 0.003	81	37.8	0.96	0.85	90	405 ± 3	41	66	0.90	422
50	0.083 ± 0.001	104	38.73	0.98	0.87	91	407 ± 2	47	84	0.91	425
100	0.064 ± 0.002	108	29.86	0. 76	0.67	92	440 ± 2	45	69	0.92	455
300	0.059 ± 0.002	152	27.54	0.69	0.62	93	406 ± 3	42	69	0.93	423
500	0.052 ± 0.003	161	24.26	0.62	0.55	94	404 ± 3	39	61	0.94	421
800	0.049 ± 0.001	166	22.86	0.58	0.52	95	440 ± 2	41	60	0.95	456
1000	0.041 ± 0.003	196	19.61	0.49	0.44	95	403 ± 3	43	59	0.95	420

**Table 5:** Kinetic parameters of OL 37 in 0.5 M H_2_SO_4_ at various concentrations of K2 at temperature of 25 °C.

Concentration (ppm)	i_corr_ (mA cm^−2^)	R_p_ Ω cm^−2^	R_mpy_	P_mm/year_	K_g_ g m^−2^ h^−1^	E (%)	-E_corr_ (mV)	b_a_ (mV dec^−1^)	-b_c_ (mV dec^−1^)	θ	-OCP (mV)
0	0.898 ± 0.020	16	419	10.63	9.45		491 ± 1	97	94	-	495
20	0.12 ± 0.001	98	56	1.42	1.26	87	402 ± 3	45	101	0.87	425
50	0.11 ± 0.002	113	51.33	1.30	1.15	87	405 ± 3	49	98	0.87	431
100	0.075 ± 0.001	103	35	0.88	0.79	92	409 ± 1	47	61	0.92	435
300	0.061 ± 0.003	159	28.46	0.72	0.64	93	407 ± 2	45	69	0.93	433
500	0.057 ± 0.002	166	26.60	0.67	0.60	94	408 ± 2	43	66	0.94	433
800	0.046 ± 0.001	179	21.46	0.54	0.48	95	405 ± 3	41	63	0.95	428
1000	0.040 ± 0.002	204	20.6	0.51	0.45	95	403 ± 3	39	61	0.95	424

**Table 6:** Kinetic parameters of OL 37 in 1 M HCl at various immersion time of K1 at temperature of 25 °C.

Immersion Time (h)	i_corr_ (mA cm^−2^)	R_p_ Ω cm^−2^	R_mpy_	P_mm/year_	K_g_ g m^−2^ h^−1^	-E_corr_ (mV)	b_a_ (mV dec^−1^)	-b_c_ (mV dec^1^)	E (%)	θ
0	0.039	324	18.2	0.46	0.41	488	76	84	95	0.95
24	0.044	229	29.86	0.75	0.67	420	97	76	93	0.94
48	0.051	228	26.6	0.68	0.60	412	88	93	74	0.93
72	0.085	174	39.66	1.01	0.91	422	114	76	89	0.90
96	0.106	159	49.46	1.25	1.18	421	115	77	88	0.88
158	0.121	142	56.46	1.43	1.27	413	114	74	86	0.86
182	0.134	109	62.53	1.58	1.41	412	115	73	83	0.84

**Table 7:** Kinetic parameters of OL 37 in 1 M HCl at various immersion time of K2 at temperature of 25 °C.

Immersion Time (h)	i_corr_ (mA cm^−2^)	R_p_ Ω cm^−2^	R_mpy_	P_mm/year_	K_g_ g m^−2^ h^−1^	-E_corr_ (mV)	b_a_ (mV dec^−1^)	-b_c_ (mV dec^1^)	E (%)	θ
0	0.042	299	20.53	0.52	0.46	422	62	95	95	0.95
24	0.030	533	14	0.35	0.32	426	66	65	96	0.96
48	0.036	427	1.86	0.40	0.36	423	82	72	96	0.96
72	0.040	408	18.66	0.47	0.42	412	78	70	95	0.95
96	0.051	411	23.8	0.60	0.54	404	68	60	94	0.94
158	0.066	264	31.05	0.79	0.71	398	74	75	92	0.92
182	0.095	205	44.33	1.12	1.01	395	74	66	88	0.88

**Table 8:** Kinetic parameters of OL 37 in 0.5 M H_2_SO_4_ at various immersion time of K1 at temperature of 25 °C.

Immersion Time (h)	i_corr_ (mA cm^−2^)	R_p_ Ω cm^−2^	R_mpy_	P_mm/year_	K_g_ g m^−2^ h^−1^	-E_corr_ (mV)	b_a_ (mV dec^−1^)	-b_c_ (mV dec^1^)	E (%)	θ
0	0.041	196	19.61	0.49	0.44	403	43	59	95	0.95
24	0.044	224	20.53	0.52	0.46	411	62	71	95	0.95
48	0.070	183	32.66	0.83	0.74	415	70	71	92	0.92
72	0.093	120	43.4	1.10	0.98	416	65	62	90	0.90
96	0.107	91	49.94	1.27	1.13	412	52	60	88	0.88
158	0.239	63	120.86	3.06	2.73	407	54	67	72	0.72

**Table 9:** Kinetic parameters of OL 37 in 0.5 M H_2_SO_4_ at various immersion time of K2 at temperature of 25 °C.

Immersion Time (h)	i_corr_ (mA cm^−2^)	R_p_ Ω cm^−2^	R_mpy_	P_mm/year_	K_g_ g m^−2^ h^−1^	-E_corr_ (mV)	b_a_ (mV dec^−1^)	-b_c_ (mV dec^1^)	E (%)	θ
0	0.040	204	20.6	0.51	0.45	401	40	61	95	0.95
24	0.064	102	29.86	0.75	0.67	487	118	82	93	0.93
48	0.098	76	45.73	1.16	1.03	477	120	81	89	0.89
72	0.22	46	102.66	2.60	2.13	497	111	90	76	0.76
96	0.35	39	163.33	17.37	3.68	460	112	91	68	0.68
158	0.44	38	205.33	5.21	4.66	499	89	142	50	0.50

**Table 10:** EIS parameters for OL 37 with K1, in 1 M HCl, at 25 °C.

Concentration (ppm)	R_S_ (ohm × cm^2^)	Q − Yo S s^−n^ cm^−2^	Q − n	R_ct_ (ohm × cm^2^)	χ^2^	E%
0	0.72	0.00518	0.74	6.5	4.255 × 10^−3^	-
20	2.911	0.000224	0.83	68	1.205 × 10^−3^	90
50	0.862	0.000202	0.88	76	2.675 × 10^−3^	91
100	1.108	0.000521	0.88	96	1.473 × 10^−3^	93
300	0.828	0.000548	0.89	156	6.908 × 10^−3^	96
500	0.7486	0.000175	0.89	122	3.758 × 10^−3^	95
800	0.7044	0.000232	0.88	177	4.248 × 10^−3^	96
1000	2.908	0.000224	0.89	207	1.175 × 10^−3^	96

**Table 11:** EIS parameters for OL 37 with K2, in 1 M HCl, at 25 °C.

Concentration (ppm)	R_S_ (ohm × cm^2^)	Q − Yo S s^−n^ cm^−2^	Q − n	R_ct_ (ohm × cm^2^)	χ^2^	E%
0	0.72	0.00518	0.74	6.5	4.255 × 10^−3^	-
20	1.939	0.000178	0.82	88	1.578 × 10^−3^	93
50	0.675	0.000196	0.88	116	3.763 × 10^−3^	94
100	5.938	0.000271	0.87	118	1.578 × 10^−3^	94
300	0.873	0.000457	0.88	108	2.125 × 10^−3^	94
500	0.7146	0.000266	0.89	111	4.394 × 10^−3^	94
800	0.5391	0.000168	0.93	125	2.140 × 10^−3^	95
1000	1.53	0.000138	0.92	239	1.397 × 10^−3^	97

**Table 12:** EIS parameters for OL 37 with K1, in 0.5 M H_2_SO_4_, at 25 °C.

Concentration (ppm)	R_S_ (ohm × cm^2^)	Q − Yo S s^−n^ cm^−2^	Q − n	R_ct_ (ohm × cm^2^)	χ^2^	E%
0	0.97	0.0063	0.76	21	4.664 × 10^−3^	-
20	1.17	0.00215	0.90	58	2.101 × 10^−3^	64
50	0.88	0.00160	0.91	86	4.484 × 10^−3^	76
100	1.43	0.00135	0.92	89	2.587 × 10^−3^	76
300	1.26	0.00122	0.91	98	2.988 × 10^−3^	79
500	0.89	0.00156	0.93	118	2.539 × 10^−3^	82
800	0.76	0.000490	0.91	138	6.575 × 10^−3^	85
1000	1.38	0.000334	0.90	153	7.815 × 10^−3^	87

**Table 13:** EIS parameters for OL 37 with K2, in 0.5 M H_2_SO_4_, at 25 °C.

Concentration (ppm)	R_S_ (ohm × cm^2^)	Q − Yo S s^−n^ cm^−2^	Q − n	R_ct_ (ohm × cm^2^)	χ^2^	E%
0	0.970	0.0063	0.76	21	4.664 × 10^−3^	-
20	1.402	0.001343	0.92	60	2.266 × 10^−3^	65
50	1.240	0.001286	0.91	74	3.292 × 10^−3^	72
100	1.168	0.001322	0.92	79	4.323 × 10^−3^	73
300	1.382	0.001383	0.89	84	3.518 × 10^−3^	75
500	1.065	0.001093	0.92	88	4.704 × 10^−3^	76
800	1.282	0.001671	0.87	99	4.345 × 10^−3^	80
1000	1.083	0.001273	0.93	162	1.478 × 10^−3^	87

**Table 14:** The values of E_a_, ΔH^0^ and ΔS^0^ for K1 and K2 on OL 37 in 1 M HCl.

Inhibitor	E_a_ (KJ mol^−1^)	ΔH^0^ (KJ mol^−1^)	ΔS^0^ (J mol^−1^ K^−1^)
K1 + HCl	45.97	43.37	−125.11
K2 + HCl	42.28	39.67	−137.76
HCl	36.65	34.16	−128.96
K1 + H_2_SO_4_	41.82	39.24	−138.63
K2 + H_2_SO_4_	49.28	46.68	−112.82
H_2_SO_4_	39.49	37.18	−122.61

**Table 15:** The values of K_ads_ and $∆G_{ads}^{{}^{\circ}}$ for K1 and K2 on OL 37 in 1 M HCl and 0.5 M H_2_SO_4_.

The System	K_ads_, M^−1^	$∆G_{ads}^{{}^{\circ}},$ KJmol^−1^	Slope	The Type of Adsorption
OL 37 + K1 + HCl	4.11 × 10^4^	−36.25	1.044	Chemisorption and physical adsorption
OL 37 + K2 + HCl	5.15 × 10^4^	−36.81	1.052	Chemisorption and physical adsorption
OL 37 + K1 + H_2_SO_4_	5.62 × 10^4^	−37.03	1.05	Chemisorptions and physical adsorption
OL 37 + K2 + H_2_SO_4_	4.36 × 10^4^	−36.37	1.048	Chemisorptions and physical adsorption

## Data Availability

The original contributions presented in this study are included in the article. Further inquiries can be directed to the corresponding authors.
